# Cervical screening with primary HPV testing or cytology in a population of women in which those aged 33 years or younger had previously been offered HPV vaccination: Results of the Compass pilot randomised trial

**DOI:** 10.1371/journal.pmed.1002388

**Published:** 2017-09-19

**Authors:** Karen Canfell, Michael Caruana, Val Gebski, Jessica Darlington-Brown, Stella Heley, Julia Brotherton, Dorota Gertig, Chloe J. Jennett, Annabelle Farnsworth, Jeffrey Tan, C. David Wrede, Philip E. Castle, Marion Saville

**Affiliations:** 1 Cancer Research Division, Cancer Council New South Wales, Sydney, New South Wales, Australia; 2 School of Public Health, Sydney Medical School, University of Sydney, Sydney, New South Wales, Australia; 3 Prince of Wales Clinical School, University of New South Wales, Sydney, New South Wales, Australia; 4 NHMRC Clinical Trials Centre, University of Sydney, Sydney, New South Wales, Australia; 5 Victorian Cytology Service, Melbourne, Victoria, Australia; 6 School of Public Health, University of Melbourne, Melbourne, Victoria, Australia; 7 Douglass Hanly Moir Pathology, Sydney, New South Wales, Australia; 8 Department of Obstetrics and Gynaecology, Melbourne Medical School, University of Melbourne, Melbourne, Victoria, Australia; 9 Department of Oncology & Dysplasia, Royal Women’s Hospital, Melbourne, Victoria, Australia; 10 Albert Einstein College of Medicine, New York, New York, United States of America; The Catalan Institute of Oncology, SPAIN

## Abstract

**Background:**

Using primary human papillomavirus (HPV) testing for cervical screening increases detection of high-grade cervical intraepithelial neoplastic lesions and invasive cancer (cervical intraepithelial neoplasia grade 2+ [CIN2+]) compared to cytology, but no evaluation has been conducted in a population previously offered HPV vaccination. We aimed to assess colposcopy referral and CIN2+ detection rates for HPV-screened versus cytology-screened women in Australia’s HPV-vaccinated population (by 2014, resident women ≤33 years had been age-eligible for HPV vaccination, with 3-dose uptake across age cohorts being about 50%–77%).

**Methods and findings:**

Compass is an open-label randomised trial of 5-yearly HPV screening versus 2.5-yearly liquid-based cytology (LBC) screening. In the first phase, consenting women aged 25–64 years presenting for routine screening at 47 primary practices in Victoria, Australia, provided a cervical sample and were randomised at a central laboratory at a 1:2:2 allocation to (i) image-read LBC screening with HPV triage of low-grade cytology (‘LBC screening’), (ii) HPV screening with those HPV16/18 positive referred to colposcopy and with LBC triage for other oncogenic (OHR) types (‘HPV+LBC triage’), or (iii) HPV screening with those HPV16/18 positive referred to colposcopy and with dual-stained cytology triage for OHR types (‘HPV+DS triage’). A total of 5,006 eligible women were recruited from 29 October 2013 to 7 November 2014 (recruitment rate 58%); of these, 22% were in the group age-eligible for vaccination. Data on 4,995 participants were analysed after 11 withdrawals; 998 were assigned to, and 995 analysed (99.7%) in, the LBC-screened group; 1,996 assigned to and 1,992 analysed (99.8%) in the HPV+LBC triage group; and 2,012 assigned to and 2,008 analysed (99.8%) in the HPV+DS triage group. No serious trial-related adverse events were reported. The main outcomes were colposcopy referral and detected CIN2+ rates at baseline screening, assessed on an intention-to-treat basis after follow-up of the subgroup of triage-negative women in each arm referred to 12 months of surveillance, and after a further 6 months of follow-up for histological outcomes (dataset closed 31 August 2016). Analysis was adjusted for whether women had been age-eligible for HPV vaccination or not. For the LBC-screened group, the overall referral and detected CIN2+ rates were 27/995 (2.7% [95% CI 1.8%–3.9%]) and 1/995 (0.1% [95% CI 0.0%–0.6%]), respectively; for HPV+LBC triage, these were 75/1,992 (3.8% [95% CI 3.0%–4.7%]) and 20/1,992 (1.0% [95% CI 0.6%–1.5%]); and for HPV+DS triage, these were 79/2,008 (3.9% [95% CI 3.1%–4.9%]) and 24/2,008 (1.2% [95% CI 0.8%–1.6%]) (*p =* 0.09 for difference in referral rate in LBC versus all HPV-screened women; *p =* 0.003 for difference in CIN2+ detection rate in LBC versus all HPV-screened women, with *p =* 0.62 between HPV screening groups). Limitations include that the study population involved a relatively low risk group in a previously well-screened and treated population, that individual women’s vaccination status was unknown, and that long-term follow-up data on disease detection in screen-negative women are not yet available.

**Conclusions:**

In this study, primary HPV screening was associated with significantly increased detection of high-grade precancerous cervical lesions compared to cytology, in a population where high vaccine uptake was reported in women aged 33 years or younger who were offered vaccination. It had been predicted that increased disease detection might be associated with a transient increase in colposcopy referral rates in the first round of HPV screening, possibly dampened by HPV vaccine effect; in this study, although the point estimates for referral rates in women in each HPV-screened group were 41%–44% higher than in cytology-screened women, the difference in referral rate between cytology- and HPV-screened women was not significant. These findings provide initial support for the implementation of primary HPV screening in vaccinated populations.

**Trial registration:**

Australian New Zealand Clinical Trials Registry ACTRN12613001207707

## Abstract 2

Karen Canfell and colleagues report results of the Compass trial; in a large Australian cohort, HPV screening is superior to pap smears for pre-cancerous lesion identification.

## Author summary

**Why was this study done?:**

- A number of countries are currently considering a transition from cytology (Pap) testing to primary HPV testing for cervical screening. This is underpinned by evidence from a number of large-scale randomised trials showing improved early detection of high-grade precancerous disease and subsequent improved long term protection against cervical intraepithelial neoplasia grade 3 (CIN3) and invasive cervical cancer in HPV-screened women compared to those screened with cytology.
- However, no prior trials of these cervical screening approaches have been conducted in a population with substantial uptake of HPV vaccination, which has now been available for a decade.

**What did the researchers do and find?:**

- Australia has a high uptake of HPV vaccination, with all women aged ≤33 years at the time of recruitment in 2014 having previously been offered vaccination. We conducted a randomised trial of 5-yearly HPV screening versus 2.5-yearly cytology screening in women aged 25–64 years presenting for routine screening in Victoria, Australia.
- We examined detected rates of high-grade precancer (CIN2+) in the baseline screening round, assessed on an intention-to-treat basis after follow-up of a subgroup of women in each arm who were referred to 12 months of surveillance based on their screening results, and after a further 6 months of follow-up for histological outcomes.
- We found that there was a significantly higher detection rate for CIN2+ in HPV-screened women (about 1.0% for HPV-screened women versus 0.1% for cytology-screened women).

**What do these findings mean?:**

- These findings provide initial confirmation of an improved performance of primary HPV screening compared to cytology screening in settings with HPV-vaccinated populations.
- These findings from Australia, the first country to adopt a national, publicly funded HPV vaccination program, are relevant to other countries considering the implications of HPV vaccination implementation on future cervical screening approaches.

## Introduction

A number of countries are currently considering a transition to primary human papillomavirus (HPV) testing for cervical screening, underpinned by evidence from a number of large-scale randomised trials and longitudinal cohort studies, which, taken together, show improved early detection of high-grade precancerous disease (cervical intraepithelial neoplasia grade 2 or 3 [CIN2/3]), lower cumulative incidence of CIN3 and invasive cervical cancer in HPV negative compared to cytology negative women, and, following treatment of detected CIN2/3, subsequent improved long-term protection against CIN3 and invasive cervical cancer in HPV-screened women compared to those screened with cytology [1–9]. However, no prior trials of primary HPV screening compared to cytology screening have been conducted in a population with substantial uptake of HPV vaccination.

Australia was the first country in the world to introduce a national HPV vaccination program. Since 2007, vaccination with quadrivalent vaccine (Gardasil, Merck/CSL) has been routinely offered to 12- to 13-year-old girls, and reported 3-dose and 2-dose coverage rates for girls aged 12–13 years in 2013 were 77% and 83%, respectively. A 2-year catch-up in females aged 12–26 years in 2007 was conducted, with women who had commenced the 3-dose course given the opportunity to complete it by the end of 2009; this achieved 3- and 2-dose coverage rates of 70% and 78% in girls aged 12–17 years, and 3- and 2-dose coverage rates of 32%–53% and 45%–59% in women aged 18–26 years [10,11]. By 2014, therefore, all women ≤33 years had been offered vaccination. The impact of vaccination has been extensively documented for several outcomes in women in their 20s, including reductions in vaccine-included HPV types, anogenital warts, and confirmed CIN2/3 lesions [12–15]. However, cervical screening will continue to be required since first-generation vaccines protect against HPV types implicated in about 70% of invasive cervical cancers. Furthermore, older women have not been offered vaccination, and currently the situation is dynamic, with the initial cohorts offered vaccination aging over time and maturing within the screening program.

Australia’s National Cervical Screening Program was established in 1991 and has involved 2-yearly conventional cytology screening in sexually active women aged 18–20 to 69 years [16]. However, in the last 5 years the program has undergone a major review, ‘renewal’, prompted by the implementation of HPV vaccination [17]. Based on systematic review of the literature and modelled evaluation of screening, it was concluded that the preferred approach to cervical screening in both unvaccinated women and cohorts offered vaccination involved 5-yearly primary HPV screening with partial genotyping for HPV16/18 in women aged 25–69 years, and discharge of HPV-negative women from screening in their early 70s. The modelled evaluation predicted reductions of approximately 20% or greater in cervical cancer incidence and mortality for this strategy compared to the current screening program [17,18]. A randomised controlled trial of primary HPV screening versus cytology-based screening, known as Compass, commenced recruitment in 2013 and is acting as a sentinel experience of primary HPV screening prior to the national screening program transition.

We aimed to use baseline screening data from the first phase of Compass (i) to determine the rate of colposcopy referral in the first round of HPV screening and to assess if this was higher than in the cytology screening arm of the trial and (ii) to assess the detected CIN2+ rate in the first screening round in HPV-screened versus cytology-screened women. Analyses were performed on an intention-to-treat basis after follow-up of women referred to 12-month surveillance based on their screening results, and after a further 6 months of follow-up for histological outcomes, and were adjusted according to whether women had been age-eligible to be offered HPV vaccination (≤33 years in 2014) (‘vaccinated cohorts’) or not age-eligible to be offered HPV vaccination (34+ years in 2014) (‘unvaccinated cohorts’).

## Methods

### Study design

Compass is a large-scale open-label randomised trial of 5-yearly HPV screening versus 2.5-yearly cytology screening coordinated at the Victorian Cytology Service (VCS), which also operates the VCS Pathology laboratory, the Victorian Cervical Cytology Registry (and now also the South Australian Cervix Screening Registry), and the National HPV Vaccination Program Register. Compass is being conducted in 2 phases. The first phase (pilot) (registration ACTRN12613001207707), for which recruitment is closed, involved a target recruitment of 5,000 women presenting for routine cervical screening. The main trial (registration NCT02328872), which is currently recruiting, has a target of 121,000 women presenting for either routine screening or follow-up management. Recruitment for the pilot was carried out in 47 primary practice and community clinics, by 247 individual clinicians, across Victoria. Human research ethics committee approval was obtained from the Alfred Hospital Ethics Committee (EC00315), the Bellberry Human Research Ethics Committee (EC00444), and the Royal Australian College of General Practitioners National Research and Evaluation Ethics Committee (NREEC 13–005). The pilot study protocol is provided in S1 Text, with a list of amendments that have been made to the protocol since the start of the trial detailed on the cover page. The majority of amendments were made in line with the development of new guidelines for the downstream management of women after screening (including post-colposcopy management) for the renewed National Cervical Screening Program and reflect the role of the trial in providing a sentinel experience for the new program. Other changes include administrative amendments, updates to data storage, and the addition of a substudy.

### Participants

**Fig 1:**
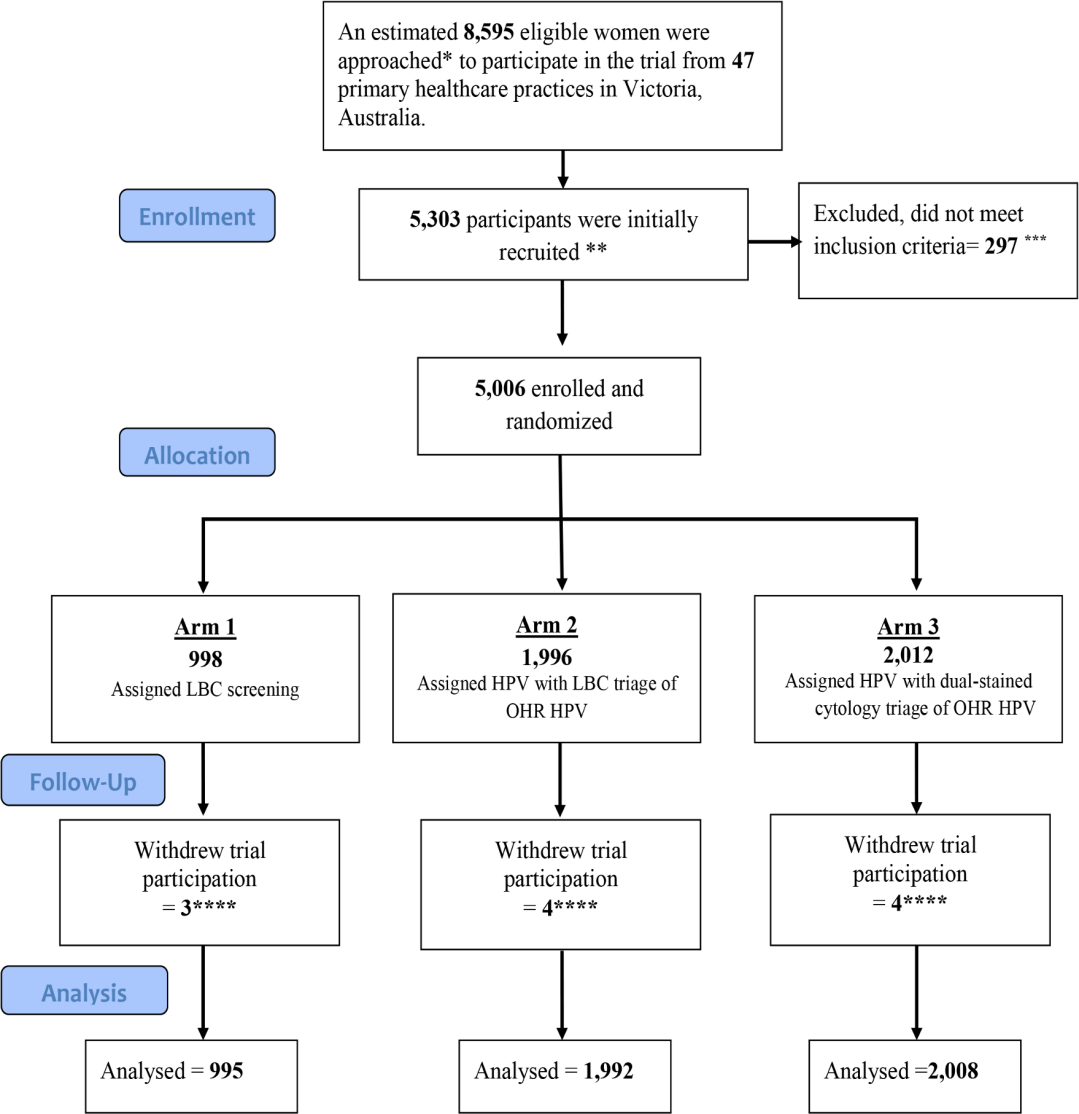
Trial profile: CONSORT 2010 flow diagram. *The number of eligible women was estimated using the total number of samples sent to the Victorian Cytology Service for cervical screening from the participating clinics. Two recruiting clinics were excluded from this estimate as their non-Compass cervical cytology samples were processed through a different laboratory and thus their precise recruitment rate cannot be obtained. **A total of 5,303 women were initially recruited. ***A total of 297 women were subsequently found to be ineligible because of their age, or because they were currently in follow-up for a previously diagnosed low-grade or high-grade abnormality, or the consent form was not received at the laboratory. ****A further 11 women subsequently withdrew from the trial; these were excluded (1 expressed concerns about the longer interval for screening, 1 did not wish to participate in verification colposcopy, 1 was anxious, 1 moved overseas and requested to be withdrawn, and 7 did not provide specific reasons). This left a total of 4,995 eligible participants for the primary outcome analysis. HPV, human papillomavirus; LBC, liquid-based cytology.

Women were recruited by medical and nurse practitioners at participating primary healthcare clinics or sexual health clinics in Victoria. The study included Australian female residents of Victoria aged 25–64 years attending for routine cervical screening or follow-up of prior unsatisfactory smear for routine screening. Exclusion criteria were as follows: previous total hysterectomy (uterus and cervix); the presence of symptoms for which cervical cancer must be excluded; currently undergoing treatment for cervical precancer or cancer; attending for follow-up of a prior cervical abnormality, including repeated ‘test-of-cure’ procedures in which the woman has not yet been discharged back to routine screening; and known pregnancy. Participating women provided written informed consent, and for these women a liquid-based cytology (LBC) sample was taken and returned to the central laboratory, VCS Pathology. Fig 1 shows the CONSORT flow diagram for the trial, from the estimated number of eligible participants approached to participate in the trial to the number of participants included in the current analysis.

### Randomisation and masking

**Fig 2:**
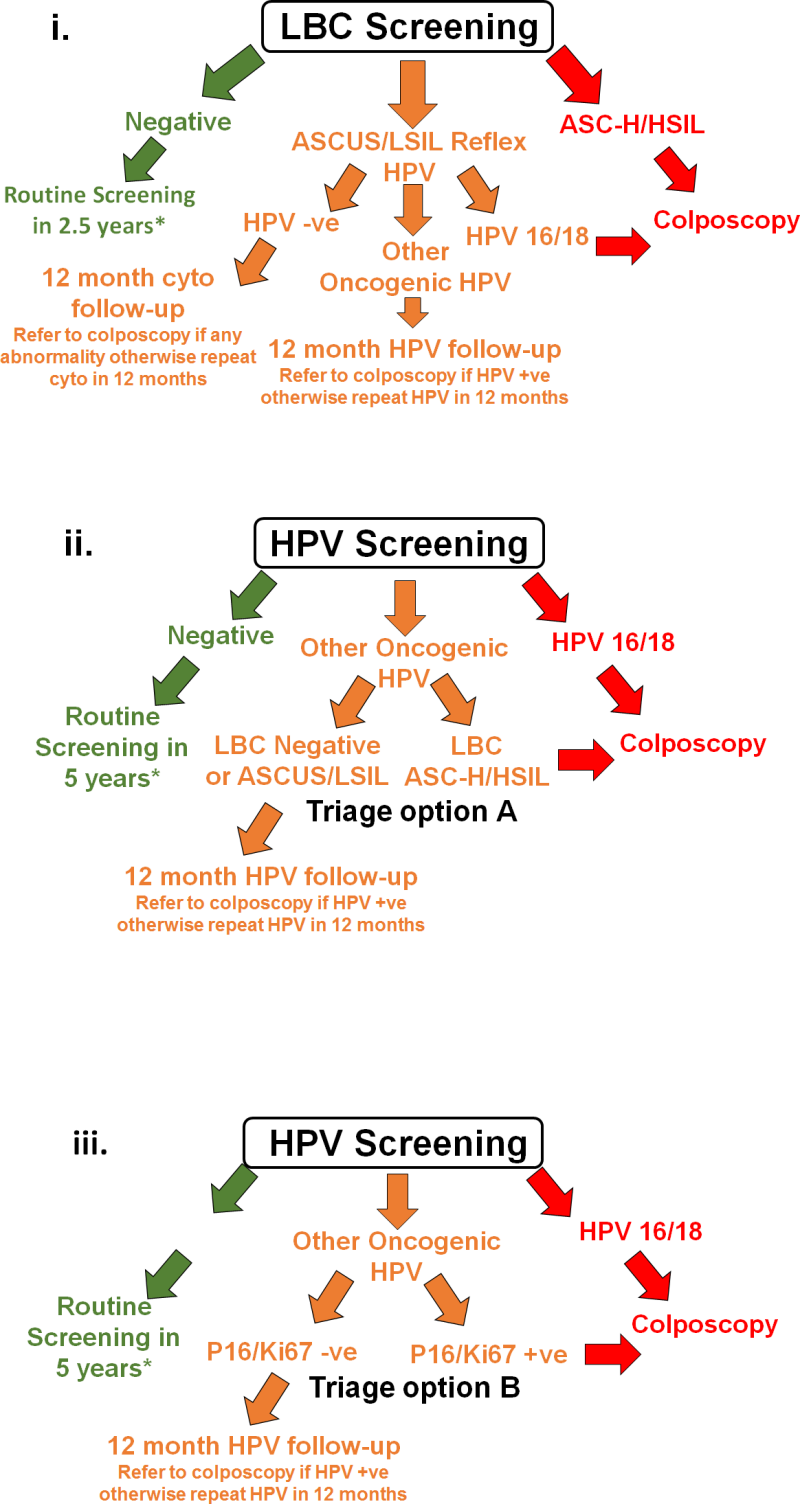
Schematic showing screening and management for each group in the trial. (i) LBC screening, (ii) HPV+LBC triage screening, and (iii) HPV+DS triage screening. *For more detailed management flowcharts, refer to S1 Text. −ve, negative; +ve, positive; ASC-H, atypical squamous cells–cannot exclude high-grade squamous intraepithelial lesion; ASCUS, atypical squamous cells of undetermined significance; cyto, cytology; HPV, human papillomavirus; HSIL, high-grade squamous intraepithelial lesion; LBC, liquid-based cytology; LSIL, low-grade squamous intraepithelial lesion.

Participants and recruiting practitioners were blinded to randomisation assignment at the time of recruitment. After receipt of the LBC sample at the centralised laboratory, participants were randomised according to a computer-generated schedule, designed by the NHMRC Clinical Trials Centre at the University of Sydney (staff generating the schedule had no involvement in other aspects of the trial). The randomisation used a minimisation procedure stratified by age group (<30 and 30+ years; an approximation of the stratification of those age-eligible for vaccination or not); no other stratification criteria were used. Minimisation is an adaptive randomisation procedure used to ensure a good balance between the 3 arms across the stratification levels, as well as overall; allocations were not determined in advance but depended on the vaccination eligibility status of the participants who had already been randomised. The randomisation allocation was available to laboratory personnel only after receipt and logging of each sample. Randomisation was at a 1:2:2 allocation ratio to (i) image-read LBC screening with HPV triage testing of low-grade cytology (‘LBC screening’); (ii) HPV screening with partial genotyping and direct referral of women with detected HPV16/18 to colposcopy and with LBC triage for other ‘high risk’ (OHR) oncogenic types (‘HPV+LBC triage’; equivalent to the new screening recommendations in Australia); or (iii) HPV screening with partial genotyping and direct referral of women with detected HPV16/18 to colposcopy and with dual-stained cytology triage testing for other oncogenic types (‘HPV+DS triage’). After randomisation, the specified screening and triage testing was conducted for each group. Fig 2 shows a schematic representation of management for women randomised to each arm of the trial; detailed management flowcharts are provided in the protocol (S1 Text). The primary practitioner was then notified of the randomisation allocation and screening result via the laboratory report, which included a recommendation for management.

### Procedures

For LBC screening and triage testing, ThinPrep (Hologic, US) was used. For HPV screening and triage testing, both HC2 (Qiagen, US) and Cobas 4800 (Roche Molecular Systems, US) technologies were used since the objectives of the study included laboratory evaluation of different HPV test technologies (see S1 Text); Cobas 4800 was used for the initial 3,104 (78%) of the total 4,003 HPV-screened women, and HC2 was used for the remainder. These different tests were not considered different treatment groups; the use of different technologies is consistent with the approach that will be used in the renewed National Cervical Screening Program, i.e., a range of clinically validated test platforms will be used for HPV testing, provided that these pre-qualify according to set criteria. For dual-stained cytology testing, CINtec PLUS technology was used (Roche Tissue Diagnostics, US), which stains for the markers p16 and Ki67 [19]. Initially, it was planned that screen-negative women randomised to cytology screening would have a screening interval of 3 years and those randomised to HPV screening would have a screening interval of 6 years. After the new 5-yearly HPV screening recommendations for Australia were announced (April 2014), ethics committee approval was obtained on 8 July 2015 to change the screening interval for the HPV-screened women in the pilot to 5 years. This change was made after all participants were recruited, and primary practitioners and enrolled participants were then informed of the change via letter. However, it should be noted that this change has no impact on the baseline screening round analysis reported here.

A total of 181 women were referred to colposcopy; attendance could be verified and histological outcomes ascertained for 177 (98%). A proportion (16%) of all women not referred to colposcopy were randomly selected and invited for verification colposcopy performed at the Royal Women’s Hospital, Melbourne. It was anticipated that this would assist in the statistical correction of potential verification bias. However, in practice, the attendance rate for invited women was low (25%), and those who attended are unlikely to represent an unbiased sample of participants. Three additional CIN2+ cases (1 in the LBC-screened group and 2 in the HPV+LBC triage group) were identified via verification colposcopy; each of these participants had screening tests with results indicating intermediate risk (therefore, if they had not been sent for verification colposcopy, they would have been followed up in 12 months). Because of the low response rate for verification colposcopy, there are substantial challenges associated with interpreting the findings, and because the current analysis focuses on referral and detection rates for screen-positive women, the verification colposcopy findings were not considered further here. More detail on the verification colposcopy is provided in S2 Text.

### Objectives

The first pre-specified primary objective was to estimate the recruitment rate and test positivity rate (summarised here as the rate of colposcopy referral); this was assessed after follow-up of the subgroup of women in each arm who were initially screen positive but based on triage test results were referred to 12 months of follow-up, and after a further 6 months of follow-up for histological outcomes (dataset closed 31 August 2016). The second primary objective was to assess the rate of detected CIN2+ in this first screening round in each group. Analysis was adjusted according to whether women had been age-eligible for HPV vaccination (in Australia, ≤33 years in 2014) or not (34+ years in 2014) (information on individual-level vaccination status was not available, because vaccination occurred many years prior to the screening test). Other objectives, assessed in a subgroup of participants and not reported here, included laboratory feasibility assessment for different HPV test technologies and the sensitivity and specificity of parallel dual-stained cytology in all HPV-positive women (for more detail, see S1 Text). Safety analyses included reported adverse events, Stage Ia2+ invasive cervical cancers (Stage Ia1 cancers were not considered here because screen-detected), and deaths in each group; event rates for the latter 2 outcomes were compared with those in the population. A proportion (10.5%) of screen-negative women in the HPV screening groups were also randomly selected for future safety monitoring with early LBC testing at 2.5 years.

### Statistical analysis

The target sample size of 5,000 was calculated in order to achieve precision in estimating the test positivity rates in women <30 and 30+ years, an approximation of the strata defined by age eligibility for vaccination as of 2013. We took into account the expected impact of HPV vaccination via estimation of the age profile of the study cohort (using historical data from the screening program), and estimated vaccine effect based on international data on effectiveness against CIN2+ (see S1 Text). This gave an expected 95% confidence interval width of 5% or less for the estimated test positivity rate in each randomisation group and stratum, taking into account the expected impact of HPV vaccination (see S1 Text). All eligible participants (excluding those who withdrew) were included in the primary and safety analyses on an intention-to-treat basis. In the analysis presented here, the Mantel–Haenszel test was used to compare the colposcopy referral rates and CIN2+ detection rates in the LBC screening arm with those in the combined HPV screening arms, and between the 2 HPV screening arms, after adjusting for HPV vaccination age eligibility. No adjustments for multiple testing were made (e.g., instead of using 0.05 as a cutoff for the *p*-values in hypothesis testing, a lower cutoff can be used [e.g., 0.01]); instead all *p*-values are quoted directly. All analyses were conducted using STATA (version 13.1).

A trial scientific advisory committee and an independent data and safety monitoring committee (IDSMC) were established. The trial was registered in the Australian New Zealand Clinical Trials Registry (ACTRN12613001207707) (retrospective registration after 1 participant was recruited), and recruitment was closed on 7 November 2014.

## Results

Of an estimated 8,595 eligible women presenting for cervical screening from 29 October 2013 to 7 November 2014, a total of 5,303 participants were initially recruited, but 297 women were subsequently found to be ineligible (according to trial inclusion/exclusion criteria) when the sample was received at the laboratory and were not randomised. Data on 4,995 participants were analysed after 11 women subsequently withdrew (Fig 1); a total of 998 were assigned to, and 995 analysed (99.7%) in, the LBC-screened group; 1,996 were assigned to and 1,992 analysed (99.8%) in the HPV+LBC triage group; and 2,012 were assigned to and 2,008 analysed (99.8%) in the HPV+DS triage group. The final estimated recruitment rate as a proportion of all women having cervical screening over the recruitment period at participating centres was 58% (range 25%–95% across centres). The randomisation allocation for eligible participants in each vaccine age-eligibility stratum is given in Table 1.

**Table 1:** Randomisation allocation by age eligibility for vaccination.

Age eligibility	LBC screening	HPV+LBC triage screening	HPV+DS triage screening	Total (%)
Offered HPV vaccination (≤33 years in 2014)	211 (21%)	418 (21%)	449 (22%)	1,078 (22%)
Not offered HPV vaccination (34+ years in 2014)	784 (79%)	1,574 (79%)	1,559 (79%)	3,917 (78%)
Total	995 (100%)	1,992 (100%)	2,008 (100%)	4,995 (100%)

Percentages by column.

DS, dual-stained; HPV, human papillomavirus; LBC, liquid-based cytology.

**Fig 3:**
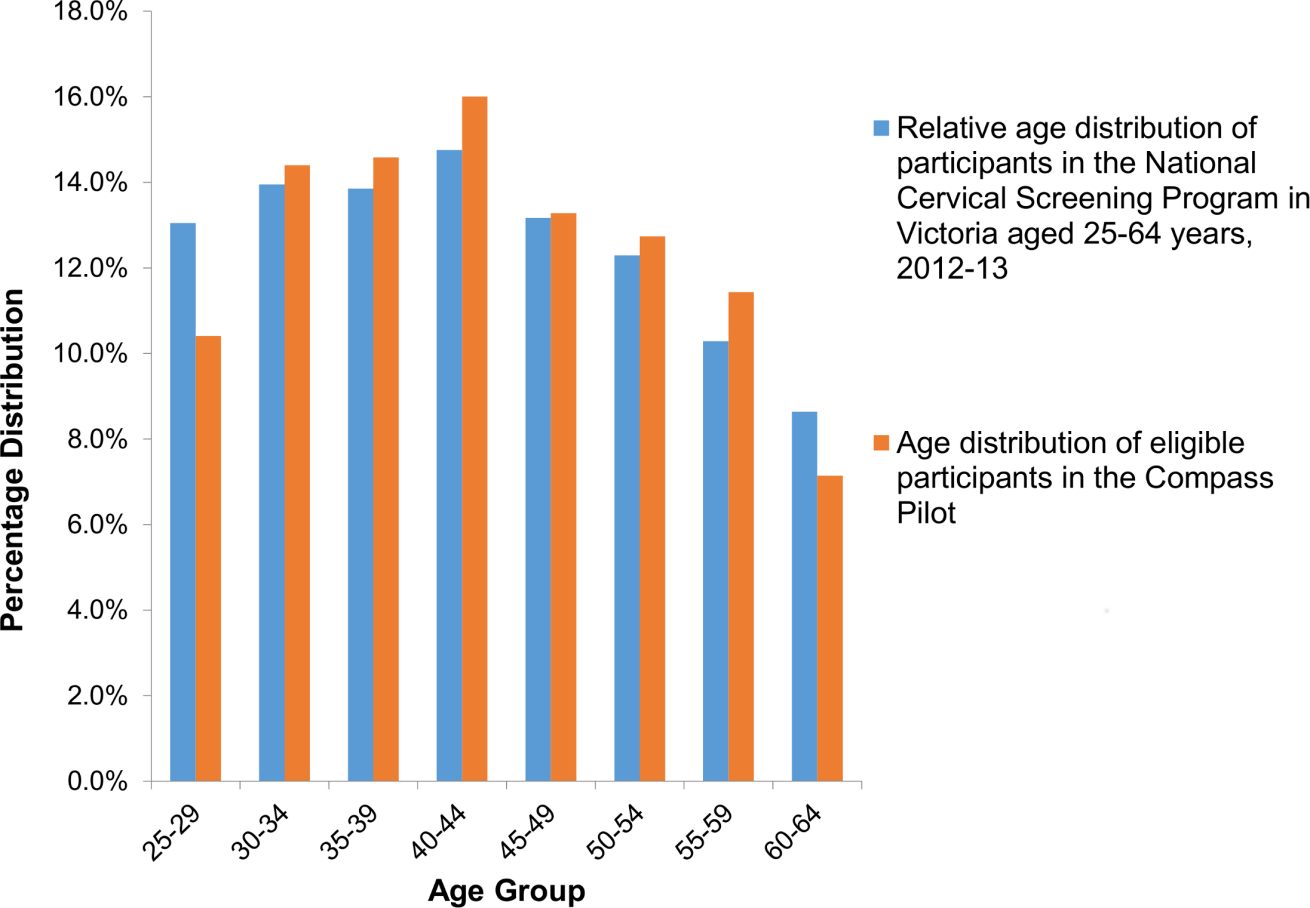
Participant age distribution, compared to those routinely attending cervical screening in Victoria. Data on women attending in Victoria for cervical screening in Australia 2012–2013 are from the Australian Institute of Health and Welfare [21].

Because this was a screening trial, available demographic information was confined to routinely collected data available from the pathology request form returned by providers and based on self-reported information; this information was compared to that from women routinely attending cervical screening in Victoria in 2012–2013 [20,21]. The women recruited to the trial were broadly age-representative of those routinely attending cervical screening (Fig 3), except that a slightly lower proportion of trial participants were aged 25–29 years (10.4% versus 13.0% of screening participants aged 25–64 years). A total of 80% of participants were Australian-born, compared to 76% in the screening population, and 91% (of 54% overall with status recorded) spoke English at home, compared to 84% (of 18% with status recorded) in the general screening population.

For the LBC screening group, the initial observed high-grade cytology rate (ASC-H/HSIL) was 0.1% (95% CI 0.0%–0.6%), and the low-grade cytology rate (ASCUS/LSIL) was 6.6% (95% CI 5.2%–8.4%); 66.7% of participants with low-grade cytology were HPV-triage negative. Due to the low rates of high-grade cytology observed, a blinded independent quality control (QC) slide re-read was performed at another laboratory routinely performing LBC, Douglass Hanly Moir Pathology, Sydney, Australia, after appropriate ethical approval was obtained and results were reported to the IDSMC. The weighted percentage agreement between the original and re-read was 96.0%, and the weighted kappa-statistic was 0.56, representing moderate agreement beyond chance. The IDSMC determined that the original reading had been appropriately and adequately performed; the main findings reported here, therefore, are from the original reading (more details on the QC re-reading process, results, and follow-up actions are provided in S3 Text). For HPV-screened women, the initial observed HPV16/18 rate was 1.2% (95% CI 0.9%–1.6%). In the HPV+LBC triage group, 1.3% (95% CI 0.8%–1.8%) of participants were HPV16/18 positive and 5.5% (95% CI 4.5%–6.6%) were OHR HPV positive; of the 109 participants who were OHR positive, 15 (13.8% [95% CI 7.9%–21.7%]) had an ASC-H/HSIL result on their reflex LBC triage test and were referred to colposcopy. In the HPV+DS triage group, 1.1% (95% CI 0.7%–1.7%) of participants were HPV16/18 positive and 6.0% (95% CI 5.0%–7.1%) were OHR HPV positive; of the 120 participants who were OHR positive, 32 (26.9% [95% CI 19.0%–35.5%]) had a positive result on their reflex dual-stained triage test and were referred to colposcopy.

**Fig 4:**
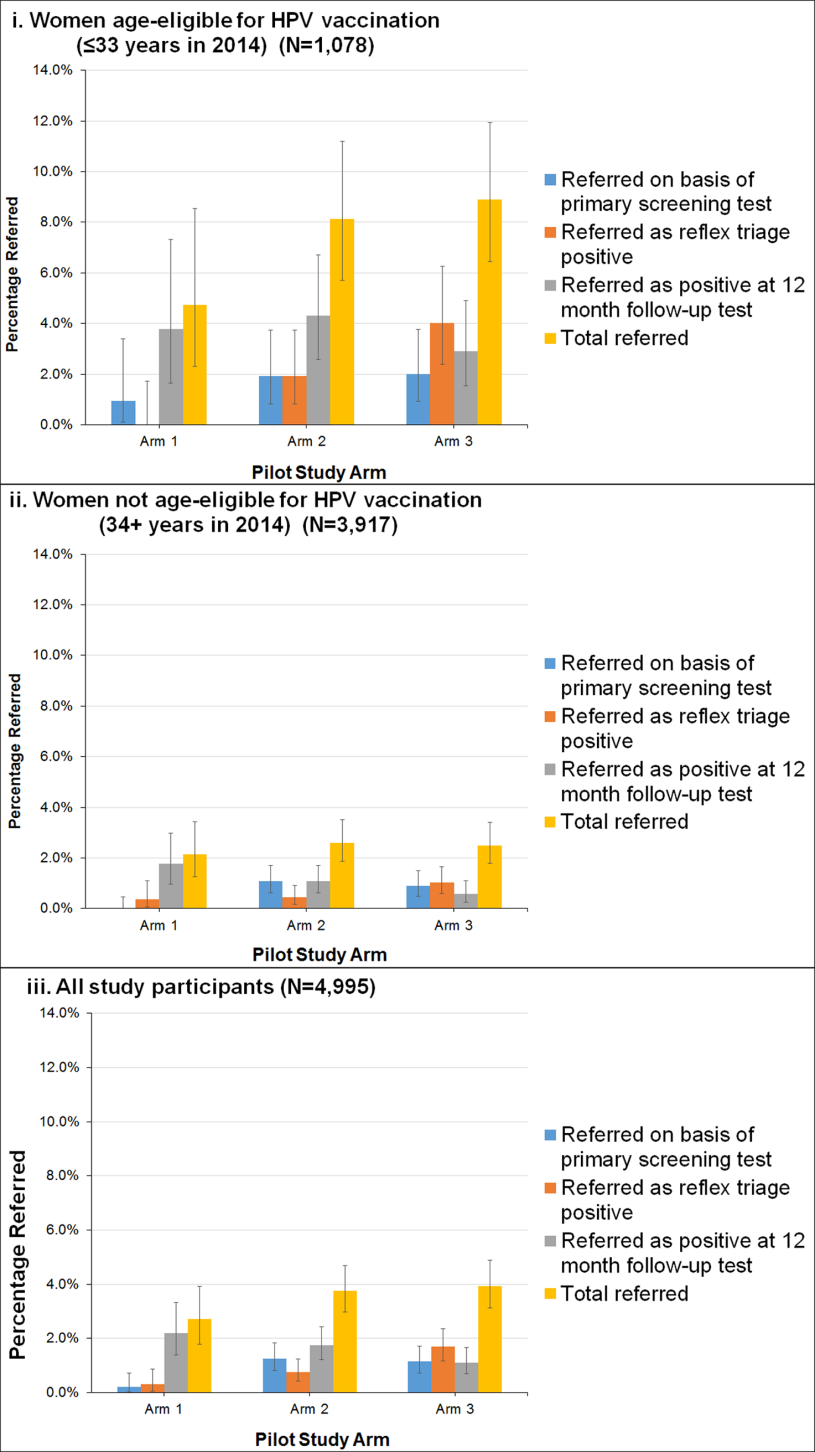
Estimated colposcopy referral rates by study group. (i) Women age-eligible for vaccination (≤33 years in 2014) (N = 1,078). (ii) Women not age-eligible for vaccination (34+ years in 2014) (N = 3,917). (iii) All study participants (N = 4,995). Bars represent 95% confidence intervals. Arm 1: LBC screening; arm 2: HPV+LBC triage screening; arm 3: HPV+DS triage screening. DS, dual-stained; HPV, human papillomavirus; LBC, liquid-based cytology.

Fig 4 shows the referral rates for each group and stratum by referral pathway (whether referred on the basis of the primary screening test, referred on the basis of a positive triage test, or detected at 12-month follow-up). The overall colposcopy referral rate in the LBC screening group was 2.7% (95% CI 1.8%–3.9%), in the HPV+LBC triage group was 3.8% (95% CI 3.0%–4.7%), and in the HPV+DS triage group was 3.9% (95% CI 3.1%–4.9%). Overall, after adjusting for HPV vaccination age eligibility, no significant difference (*p =* 0.09) was found between the referral rates in the LBC-screened and all HPV-screened women (this was also the case when the LBC QC re-reading results were used: *p =* 0.17 between LBC- and HPV-screened women). For the HPV+DS triage group, the percentage of OHR HPV initially referred as a result of a dual-stained positive result was approximately double that referred on the basis of a high-grade LBC result in the HPV+LBC triage group, but after the 12-month follow-up was taken into account, no significant difference was detected between the rates in the 2 HPV screening groups overall (*p* = 0.89) (Fig 4). The initial colposcopy referral rates in each group (i.e. referred on basis of primary screening or reflex triage test) were also compared to a historical reference rate of 1.5%, which was the colposcopy referral rate observed in women aged 20–69 years in Victoria in 2013. The initial rate in the LBC screening group was significantly lower than the reference rate (*p =* 0.02), the rate in the HPV+DS triage group was significantly higher than the reference rate (*p* < 0.001), and the rate in the HPV+LBC triage group was not significantly different from the reference rate (*p =* 0.06). However, this comparison with historical data should be interpreted cautiously given that it included initial referrals only and the trial did not include women who were currently under active surveillance for a previous abnormality, and the historical data reflects a mix of different indications for referral in a well screened population.

Table 2 shows the confirmed CIN2+ and CIN3+ rates in each group. The overall CIN2+ rates in the LBC screening, HPV+LBC triage, and HPV+DS triage groups were 0.1%, 1.0%, and 1.2%, respectively, and the corresponding CIN3+ rates were 0.1%, 0.7%, and 0.8%, respectively. A significant difference (*p =* 0.003) was identified between the CIN2+ rates in the LBC-screened versus all HPV-screened women combined, but no significant difference was identified between overall CIN2+ rates in the 2 HPV-screened groups (*p =* 0.62) after adjusting for HPV vaccination eligibility. The overall positive predictive values (PPVs) for CIN2+ and CIN3+ by referral mechanism are shown in Table 3. In the HPV-screened women, for the subgroup of women immediately referred as HPV16/18 positive, the point estimates of the PPVs for CIN2+ and CIN3+ were 40.0% and 32.0% in the HPV+LBC triage group and 39.1% and 30.4% in the HPV+DS triage group. The overall PPVs for CIN2+ and CIN3+, considering both primary and triage referral mechanisms and 12-month follow-up, were 3.7% and 3.7% for LBC screening, 26.7% and 17.3% for HPV+LBC triage, and 30.4% and 21.5% for HPV+DS triage.

**Table 2:** Case numbers and rates for detected CIN2+ and CIN3+, by age eligibility for vaccination.

Group	CIN2+	CIN3+
***LBC screening (N = 995)***		
**Age-eligible for HPV vaccination (*N =* 211)**		
Rate	1	1
Percent	0.5%	0.5%
95% CI	0.0%–2.6%	0.0%–2.6%
**Not age-eligible for HPV vaccination (*N =* 784)**		
Rate	0	0
Percent	0.0%	0.0%
95% CI	0.0%–0.5%	0.0%–0.5%
**Total (*N =* 995)**		
Rate	1	1
Percent	0.1%	0.1%
95% CI	0.0%–0.6%	0.0%–0.6%
***HPV+LBC triage screening (N = 1*,*992)***		
**Age-eligible for HPV vaccination (*N =* 418)**		
Rate	11	9
Percent	2.6%	2.2%
95% CI	1.3%–4.7%	1.0%–4.0%
**Not age-eligible for HPV vaccination (*N =* 1,574)**		
Rate	9	4
Percent	0.6%	0.3%
95% CI	0.3%–1.1%	0.1%–0.6%
**Total (*N =* 1,992)**		
Rate	20	13
Percent	1.0%	0.7%
95% CI	0.6%–1.5%	0.3%–1.1%
***HPV+DS triage screening (N = 2*,*008)***		
**Age-eligible for HPV vaccination (*N =* 449)**		
Rate	13	9
Percent	2.9%	2.0%
95% CI	1.6%–4.9%	0.9%–3.8%
**Not age-eligible for HPV vaccination (*N =* 1,559)**		
Rate	11	8
Percent	0.7%	0.5%
95% CI	0.4%–1.3%	0.2%–1.0%
**Total (*N =* 2,008)**		
Rate	24	17
Percent	1.2%	0.8%
95% CI	0.8%–1.8%	0.5%–1.4%

CIN, cervical intraepithelial neoplasia; DS, dual-stained; HPV, human papillomavirus; LBC, liquid-based cytology.

**Table 3:** PPVs for CIN2+ and CIN3+, by allocation group and referral pathway.

CIN grade and group	Referral pathway			
	Referred on basis of primary screening test*	Referred as reflex triage positive*	Referred to colposcopy for the first time at 12-month follow-up test	All referrals
***CIN2+***				
**LBC screening (*N =* 995)**				
PPV	0/2	0/3	1/22	1/27
Percent	0.0%	0.0%	4.5%	3.7%
95% CI	0.0%–84.2%	0.0%–70.8%	0.1%–22.8%	0.0%–19.0%
**HPV+LBC triage screening (*N =* 1,992)**				
PPV	10/25	8/15	2/35	20/75
Percent	40.0%	53.3%	5.7%	26.7%
95% CI	21.1%–61.3%	26.6%–78.7%	0.7%–19.2%	17.1%–38.1%
**HPV+DS triage screening (*N =* 2,008)**				
PPV	9/23	13/34	2/22	24/79
Percent	39.1%	38.2%	9.1%	30.4%
95% CI	19.7%–61.5%	22.2%–56.4%	1.1%–29.2%	20.5%–41.8%
***CIN3+***				
**LBC screening (*N =* 995)**				
PPV	0/2	0/3	1/22	1/27
Percent	0.0%	0.0%	4.5%	3.7%
95% CI	0.0%–84.2%	0.0%–70.8%	0.1%–22.8%	0.0%–19.0%
**HPV+LBC triage screening (*N =* 1,992)**				
PPV	8/25	4/15	1/35	13/75
Percent	32.0%	26.7%	2.9%	17.3%
95% CI	14.9%–53.5%	7.8%–55.1%	0.0%–14.9%	9.6%–27.8%
**HPV+DS triage screening (*N =* 2,008)**				
PPV	7/23	9/34	1/22	17/79
Percent	30.4%	26.5%	4.5%	21.5%
95% CI	13.2%–52.9%	12.9%–44.4%	0.1%–22.8%	13.1%–32.2%

Overall, 181 women were referred to colposcopy; histological outcomes could be ascertained for 177 (98%), which are reported here; those for whom no histology result was available were assumed to have not received biopsy and were thus considered as screen positive but without detected CIN2+ in this intention-to-treat analysis.

*Each of these groups includes 2 women referred to colposcopy initially who had a histological result of a grade lower than CIN2 and were then referred for follow-up at 12 months, and in whom CIN2+ was then detected at the 12-month colposcopy. See protocol (S1 Text).

CIN, cervical intraepithelial neoplasia; DS, dual-stained; HPV, human papillomavirus; LBC, liquid-based cytology; PPV, positive predictive value.

In young women, previously age-eligible for vaccination, the overall colposcopy referral rate and detected CIN2+ rate (including 12-month follow-up) for LBC screening were 10/211 (4.7% [95% CI 2.3%–8.5%]) and 1/211 (0.5% [95% CI 0.0%–2.6%]), respectively; for HPV+LBC triage these were 34/418 (8.1% [95% CI 5.7%–11.2%]) and 11/418 (2.6% [95% CI 1.3%–4.7%]), and for HPV+DS triage these were 40/449 (8.9% [95% CI 6.4%–11.9%]) and 13/449 (2.9% [95% CI 1.6%–4.9%]) (*p =* 0.81 between HPV screening groups; *p =* 0.05 for difference in detected CIN2+ in cytology-screened women versus all HPV-screened women). In older women, age-ineligible for vaccination, the colposcopy referral rate and detected CIN2+ rate for cytology screening were 17/784 (2.2% [95% CI 1.3%–3.4%]) and 0/784 (0.0% [95% CI 0.0%–0.5%]), respectively; for HPV+LBC triage screening these were 41/1,574 (2.6% [95% CI 1.9%–3.5%]) and 9/1,574 (0.6% [95% CI 0.3%–1.1%]), and for HPV+DS triage screening these were 39/1,559 (2.5% [95% CI 1.8%–3.4%]) and 11/1,559 (0.7% [95% CI 0.4%–1.3%]) (*p =* 0.64 between HPV screening groups; *p =* 0.02 for difference in detected CIN2+ in cytology-screened women versus all HPV-screened women).

Follow-up for adverse events was done to 31 August 2016. Two deaths were reported in follow-up for the HPV+LBC triage group (0.1% of participants in this group), and 2 deaths were reported for the HPV+DS triage group (0.1% of participants in this group), but all 4 were classified as unrelated to the trial by the IDSMC. No other serious adverse events were reported. Adverse events were reported in 1 participant in the LBC screening group (0.1%) and 1 participant in the HPV+LBC triage group (0.05%); these adverse events were related to management of communication of screening results. No invasive cervical cancer cases (Stage Ia2 and above) were reported in follow-up (it should be noted that 1 Stage Ia1 microinvasive cancer was detected after the initial screen in the HPV+LBC triage group, but this is not an adverse event since the invasive cancer was appropriately screen-detected). See S4 Text for more detail on adverse events.

## Discussion

Compass is, to our knowledge, the first randomized trial of cervical screening conducted in an extensively HPV-vaccinated population and one of the first trials of HPV screening using a partial genotyping strategy in which women with the highest risk HPV infections (HPV16 and HPV18) are directly referred to colposcopy. In the current study, we have demonstrated acceptably high rates of trial participation and, by implication, high rates of acceptability of primary HPV screening at longer intervals. In this first analysis, we found an increased detection rate of high-grade precancerous disease (CIN2+) in the first round of screening for HPV-screened compared to cytology-screened women, and for the first time, to our knowledge, we have demonstrated that this increased detection rate is also seen in a population for whom the younger cohort had been offered HPV vaccination, with high levels of vaccine uptake.

These findings for the relative benefits of HPV versus cytology screening are consistent with those from earlier trials conducted in unvaccinated populations. Meta-analysis of 8 randomised trials conducted mainly in Europe and North America found that in the first round of screening, the relative sensitivity of HPV versus cytology for the detection of CIN2+ was 1.27 (95% CI 1.06–1.52) [22]. Longitudinal follow-up of trials in unvaccinated populations has consistently demonstrated that this increased detection of CIN2/3, which is then treated, leads to longer term protection against the development of CIN3 and invasive cervical cancer. A pooled analysis of 4 European trials demonstrated that rates of invasive cervical cancer in HPV-screened women are significantly and substantially lower than those in cytology-screened women at about 5–6 years of follow-up [1]. Our demonstration that this higher detection rate for CIN2+ in the first screening round is observed in HPV-screened women even in a vaccinated population suggests that HPV screening will also provide increased long-term protection against cervical cancer in this context.

Our estimates of the PPV of HPV16/18 test positivity for the concurrent detection of CIN2+ (up to about 27–30% when all referrals are considered) are broadly consistent with the baseline screening round estimates for the predominantly unvaccinated women in the ATHENA trial. However, because HPV infection is a predictor of future, as well as current, CIN2+ development, the PPV for CIN2+ in ATHENA rose over the duration of 3 years of trial follow-up [8]. For example, for HPV16, the PPV for CIN2+ in ATHENA was >20% at baseline, rising to about 30% over 3 years. In the Compass trial, and in the new HPV-based screening program for Australia, women referred to colposcopy with HPV16/18 infection who do not have a detected CIN2+ lesion are referred to ongoing 12-month surveillance and repeat colposcopy referral if they remain HPV16/18 positive [25]; hence, there are ongoing opportunities to detect CIN2+ in this group of higher risk women. Therefore, further follow-up analysis from this study and the Compass main trial will provide important information about the long-term protective effect of HPV screening in relation to that of cytology screening. The Compass main trial is powered to detect a difference in the longitudinal rates of CIN3 and invasive cancer (CIN3+) at 5 years between cytology-screened and HPV-screened women as the primary outcome.

The majority of prior trials of HPV screening have involved testing for a pool of oncogenic types and triaging all HPV-positive women using cytology. The current analysis also provides, to our knowledge for the first time in a randomised trial, key findings for colposcopy referral rates in the first round of screening when using a partial genotyping strategy for HPV screening starting at age 25 years in a vaccinated population. We found that the initial colposcopy referral rate in women screened with HPV+LBC triage testing was not significantly higher than a historical reference rate (1.5%); initial referrals would have been expected to be substantially higher for partial genotyping-based HPV screening in the pre-vaccination era, when rates of HPV16/18 infection in young women were much higher [26]. An earlier US-based trial of HPV screening with partial genotyping starting at age 25 years conducted in a predominantly unvaccinated population, ATHENA, found that rates of detected HPV16/18 infection were almost 6% in women aged 25–34 years [24], compared to approximately 2% as observed in the current study for a similar age group (Fig 4). However, in relation to the interpretation of our findings for colposcopy referral rates, it should be noted that in this initial study we enrolled only women who were presenting for routine screening, and therefore it is likely that the initial referral and abnormality rates observed will be somewhat lower than those in the program overall, where referrals from women presenting for follow-up surveillance testing are also considered. The next phase of Compass has been designed to address these issues since, in the Compass main trial, recruitment of women in follow-up is also being performed.

When we considered the additional colposcopy referrals after 12 months follow-up testing, our finding of a difference in the overall colposcopy referral rate between cytology-screened and HPV-screened women was not significant, although the point estimates for referral rates in women in each HPV-screened group were 41%–44% higher than in cytology-screened women. A recent modelled analysis of anticipated health resource utilisation after the transition from cytology screening to primary HPV screening in Australia has suggested that colposcopy referrals could transiently increase by around 50% in the first round of HPV screening [27], which is broadly consistent with our results. Evidence from trials and modelling [22,27] suggests that this increased referral rate for HPV screening in the first round results in large part from an increased sensitivity for CIN2+ and thus represents increased detection of prevalent precancerous disease; it is expected that colposcopy referral rates after the transition to the HPV screening program will decrease in later screening rounds, which will then be detecting mainly incident disease. Although, for the reasons cited above, careful interpretation of our findings for referral rates is required, the current results do provide important initial information on the health service implications of implementing partial genotyping strategies for HPV16/18 in a population vaccinated against these same genotypes. This is important because the partial genotyping screening strategy allows for a highly integrated approach to screening and vaccination; the same screening and management pathways can be used independently of a woman’s vaccination status, which will not necessarily be known at the time of screening [23].

Our analysis has some limitations. We did not have information on individual women’s vaccination status, because vaccination occurred several years prior to screening; future work will involve post hoc linkage of data on Compass participants to Australia’s National HPV Vaccination Program Register to determine vaccination timing and dose status for individual participants. Our findings reflect a previously well-screened population and are most directly applicable to settings in which relatively high coverage has already been achieved for cervical screening. Since we excluded women currently under follow-up surveillance after treatment, and since women are more likely to be treated at a younger age, it is possible that this might have had an impact on the study population. However, it should be noted that the age distribution of our study population was broadly representative of the age distribution of women presenting for cervical screening in Victoria, although we did have a slightly lower proportion aged 25–29 years. As discussed above, more information will be obtained from the next phase of the trial, in which recruitment of women in follow-up is also being performed. Our finding of a low rate of high-grade cytology and detected CIN2+ in the LBC screening group was somewhat unexpected, but likely reflects the lower risk population recruited, which included only those presenting for routine screening (although participating women may have had a prior abnormality in the past, we did not recruit those currently under surveillance follow-up for a previous low-grade abnormality or prior CIN2/3 treatment). A complete QC re-read at an independent laboratory identified no additional histologically confirmed CIN2+ disease, and the rate of low-grade abnormalities (about 7%) was consistent with rates routinely observed in the laboratory. It is possible that this low rate of disease detection by cytology reflects the impact of vaccination against HPV16/18 in the population, and future analysis of data from the Compass main trial is expected to provide more detailed estimates on the cytology rates by age stratum. Recent analysis of Victorian data on detected CIN2+ rate with the current cytology-based program in women aged 25–29 years suggests a rate of about 2% in 2011, decreasing to about 1.5% in 2014 [15], and this decrease has been attributed to vaccine impact. In our study, in young women age-eligible for vaccination (≤33 years in 2014), the overall detected CIN2+ rate (including CIN2+ detected at 12-month surveillance) for LBC screening in 2014–2015 was 0.5%; this lower point estimate compared to that in the Victorian program data (general population presenting for screening) is consistent with the lower risk profile expected in the study group. However, it should be noted that the 95% confidence interval for the trial estimate extends up to 2.6%, which is consistent with the observed data in the Victorian population. In Victoria, 2014 rates of CIN2+ in women >40 years in 2014 were about 0.3% [15], which is also broadly consistent with the trial estimate of 0.0% (95% CI 0.0–0.5) for cytology screening in the older cohort age-ineligible for vaccination. Over time, it is expected that the ongoing impact of vaccination on younger cohorts will work to reduce CIN2+ rates further and to extend the vaccination effect to older ages as vaccinated cohorts mature.

We have reported initial results on the performance of dual-stained cytology using p16/Ki67 in the direct management of the subgroup of HPV-positive women who have other high-risk (not HPV16/18) infections. Compared to using LBC with a high-grade cytology threshold as a triage test for referral, we found an approximate doubling of initial referrals when using dual-stained cytology as a triage test. However, once the additional cases detected at 12-month surveillance of triage-negative women were taken into account, we did not detect a significant difference between the 2 HPV triaging strategies, which had similar overall referral rates and PPVs. Further analysis from the Compass main trial is expected to provide more detailed data on the accuracy of dual-stained cytology for triaging HPV-positive women in screening programs. Future results will also yield detailed information on the loss to follow-up experienced for women referred to 12-month surveillance, which might act to decrease the “effective” relative performance of LBC compared to dual-stained triage testing, since a higher proportion of disease was found during surveillance after LBC triage. The performance of dual-stained cytology has future implications for workforce planning, since there is the potential to automate dual-stained cytology, and as countries transition to longer interval HPV screening, there are likely to be significant fluctuations in test volumes for triage tests, as well as for the numbers of primary screening tests [27]. The automation of the triage testing process, as well as of the primary screening step, will therefore be of potential importance in the practical implementation of large-scale HPV-based screening programs.

Because the Compass trial is acting as a sentinel experience for program transition, the findings reported here are directly informing several aspects of the implementation of Australia’s new screening program, which is due to transition to primary HPV screening on 1 December 2017. For example, the HPV test positivity rates reported here have underpinned the design of new quality assurance processes that describe the expected rates to be observed in individual laboratories. The trial will also underpin national safety monitoring processes for the new screening program, since it will continue to provide early information on how HPV test positivity rates, colposcopy referral rates, and detected abnormality rates change over time as the impact of HPV vaccination continues to change outcomes in the screening program as vaccinated cohorts age. Our findings also have relevance for other countries currently transitioning to primary HPV screening in the context of vaccination. These results represent, to our knowledge, the first demonstration of partial genotyping strategies for primary HPV screening in a vaccinated population and provide the first evidence that HPV screening is associated with increased detection of cervical precancerous lesions in vaccinated, as well as unvaccinated, populations.

## Supporting information

S1 Text Trial protocol. (PDF)

S2 Text Verification colposcopy recruitment rates. (PDF)

S3 Text Findings from the quality control re-read for the LBC screening group. (PDF)

S4 Text Adverse event report. (PDF)

S5 Text Compass pilot CONSORT statement. (PDF)

S6 Text Compass pilot ethics approval. (PDF)

## Abbreviations

ASC-H: atypical squamous cells–cannot exclude high-grade squamous intraepithelial lesion
ASCUS: atypical squamous cells of undetermined significance
CIN: cervical intraepithelial neoplasia
DS: dual-stained
HPV: human papillomavirus
HSIL: high-grade squamous intraepithelial lesion
IDSMC: independent data and safety monitoring committee
LBC: liquid-based cytology
LSIL: low-grade squamous intraepithelial lesion
PPV: positive predictive value
QC: quality control
VCS: Victorian Cytology Service
