# Supplementary material for: Cervical screening with primary HPV testing or cytology in a population of women in which those aged 33 years or younger had previously been offered HPV vaccination: Results of the Compass pilot randomised trial
Source: PLoS Med. 2017 Sep 19;14(9):e1002388. doi: 10.1371/journal.pmed.1002388 (PMC5604935; doi:10.1371/journal.pmed.1002388)
Supplement: S1 Text — (PDF) [file pmed.1002388.s001.pdf]

Appendix to the manuscript:

Cervical screening with primary HPV testing or cytology in a population of women in which those aged 33 years or younger were offered vaccination: Results of the Compass pilot randomized trial

Karen Canfell,[1,2,3*] Michael Caruana, [1] Val Gebski, [4] Jessica Darlington-Brown MPH, [1] Stella Heley, [5] Julia Brotherton, [5,6] Dorota Gertig, [5,6,] Chloe J. Jennett, [1] Annabelle Farnsworth, [2,7] Jeffrey Tan, [8,9] C. David Wrede, [8,9] Philip E. Castle, [10] Marion Saville, [5,6]

[1]Cancer Research Division, Cancer Council New South Wales, Sydney, New South Wales, Australia

[2]School of Public Health, Sydney Medical School, University of Sydney, Sydney, New South Wales, Australia

[3]Prince of Wales Clinical School, The University of New South Wales, Sydney, New South Wales, Australia

[4]NHMRC Clinical Trials Centre, University of Sydney, Sydney, New South Wales, Australia

[5]Victorian Cytology Service Ltd., Melbourne, Victoria, Australia *(affiliation during time of the work for DG)*

[6]School of Public Health, University of Melbourne, Melbourne, Victoria, Australia

[7]Douglas Hanly Moir Laboratory, Sydney, New South Wales, Australia

[6]Department of Obstetrics and Gynaecology, University of Melbourne, Melbourne, Victoria, Australia

[9]Department of Oncology & Dysplasia, The Royal Women’s Hospital, Melbourne, Victoria, Australia

[10]Albert Einstein College of Medicine, Bronx, New York, The United States of America.

Appendix Section S1.

Protocol for Compass Pilot Study: A pilot for a randomised controlled trial of primary HPV DNA testing for cervical cancer screening in Australia.

Notes:

(1) Standard Bethesda terminology is used for cytology classification in the main manuscript. However the terminology used in this protocol is that of the Australian Modified Bethesda System in which the term 'pLSIL' is used for ASC-US; 'dLSIL' for LSIL; 'pHSIL' for ASC-H and 'pHSIL' for HSIL, when mapped to standard Bethesda terminology.

(2) The protocol-specified objectives of the pilot study were to assess (i) participant acceptance of the randomisation process and use of longer routine screening intervals via quantification of the recruitment rate; (ii) confirm the operational feasibility of laboratory processing procedures for two alternative HPV test platforms (HC2 [Qiagen, MD USA] and Cobas 4800 [Roche Molecular Systems, CA, USA]); (iii) to assess test positivity rates for the primary screening test in each arm; and (iv) to estimate the sensitivity and specificity of dual-stained cytology testing [CINtec Plus, Roche/Ventana, CA, USA] in the context of a positive HPV result. In this manuscript, we report on findings for the baseline screening round after completion of six month follow-up for histology outcomes; this includes analysis for Objectives (i) and (iii); analyses for Objectives (ii) and (iv) are forthcoming.

Protocol for Compass Pilot Study:

A pilot for a randomised controlled trial of primary HPV DNA testing for cervical cancer screening in Australia

A trial in both HPV-vaccinated and unvaccinated women to assess 1) outcomes after primary HPV screening compared to image-read cytology screening and 2) the optimal management strategy for HPV-positive women after primary HPV screening.

Co-PIs: Karen Canfell and Marion Saville

VERSION 2.0 7th October 2016

Summary of changes		
**Version**	**Date**	**Amendments made**
**Version 1.5**	12th Feb 2013	The first HREC approved and utilized protocol.
**Version 1.6**	21st Aug 2013	Administrative changes and addition of Proposed Main trial objectives added.
**Version 1.7**	26th Mar 2015	Participant follow up was changed from 3 to 2.5 years for LBC and 6 to 5 years for Primary HPV testing. This is in line with the renewed national cervical screening program.
**Version 1.8**	6th Aug 2015	Linear Array sub-study was added, administrative changes made.
**Version 1.9**	15th Oct 2015	Data storage information was changed, in line with CCNSW organization protocol, this change specified that de-identified study data would be stored at the Azure data centres.
**Version 2.0**	7th Oct 2016	Management process flow charts for each of the study arms was updated in line with the renewed National Cervical Screening Program and National Pathology Accreditation Advisory Council guidelines

Table of Contents

1. Glossary of Terms 6
2. Summary 10
3. Introduction 11
   3.1 Current status of cervical screening in Australia 11
4. Objectives 15
   4.1 Pilot Study Objectives 15
   4.2 Proposed Main Trial Objectives 15
5. Methods 16
   5.1 Qualitative work 16
   5.2 Recruitment rates (Objective 1) 16
      5.2.1 Analysis Plan 17
   5.3 Operational Feasibility (Objective 2) 17
      5.3.1 Analysis Plan 18
   5.4 Test positivity rates (Objective 3) 18
      5.4.1 Analysis Plan 18
   5.5 Dual Stained (DS) Cytology Sub-study (Objective 4) 18
      5.5.1 Analysis Plan 19
6. Pilot Study Design 21
   6.1 Design 21
   6.2 Sample size 21
   6.3 Participants 27
   6.4 Interventions 27
   6.5 Screening and triage test technologies 31
   6.6 Biobanking 31
7. Recruitment and Clinical Management 32
   7.1 Coordinating Centres and Study Investigators 32
   7.2 Informed consent procedures 32
   7.3 Randomisation procedures 33
   7.4 Laboratory Processes 33
   7.5 VCS Pathology Report Processes 33
   7.6 Verification colposcopy 33
   7.7 Histopathology 34
   7.8 Safety monitoring-HPV Screening (Arms 2 &3) 35
   7.9 Opting-out of the trial 36
   7.10 Transition from Pilot to Main Trial 36
   7.11 Use of trial data for cost-effectiveness modelling 36
   7.12 Victorian Cervical Cytology Registry (VCCR) Follow-up 37
8. Study Monitoring 37
   8.1 Identifying, Recording and Managing Adverse Events 38
      8.1.1 Definition of Adverse Events (AE) and Serious Adverse Events (SAE) 38
      8.1.2 Documenting and Recording AEs or SAEs 38
      8.1.3 Evaluating AEs and SAEs 39
      8.1.4 Assessment of Causality 39
      8.1.1 Practitioner Reporting of AE or SAE 39
      8.1.2 Reporting, Filing and Electronic Recording 39
      8.1.3 Reporting to the Therapeutic Goods Administration 40
   8.2 Quality Assurance and Control 40
      8.2.1 VCS Inc. Operational Quality Assurance and Control 41
      8.2.2 Laboratory Quality Assurance 41
   8.3 Data Safety and Monitoring Board (IDSMC) 41
   8.4 Data Storage and Security 41
      8.4.1 Data Transfer, Storage and Management 42
   8.5 Data linkage 42

9. Governance 42
9.1 Scientific Advisory Committee 42
9.2 Trial Reporting 43
9.3 Trial Registration and Protocol Availability 43
9.4 Human Research Ethics Committee approval 43
9.5 Protocol amendments 43
9.6 Insurance and Indemnification 43
10. References 46

1. Glossary of Terms

Term	Definition
A	
ASCUS	In the standard US Bethesda System, a category of atypical squamous cells of undetermined significance: The nature of the abnormality is uncertain or unequivocal. Equivalent to pLSIL in the Australian Modified Bethesda System.
B	
Biopsy	Removal of a small piece of tissue for laboratory examination to determine the presence or extent of disease.
C	
Cervical Intraepithelial Neoplasia (CIN)	Refers to abnormal changes in the cells on the surface of the cervix that are seen underneath a microscope (i.e. histologically-confirmed).<br>CIN 1 -- mild dysplasia<br>CIN 2 -- moderate dysplasia<br>CIN 3 -- severe dysplasia to carcinoma in situ<br>(The term CIN 2+ refers to CIN 2, 3, or invasive cervical cancer; CIN3+ refers to CIN 3 or invasive cervical cancer).
Colposcopy	The examination of the cervix and vagina with a magnifying instrument called a colposcope, to check for abnormalities.
CONSORT Statement (CONsolidated Standards of Reporting Trials)	A checklist which aims to improve the reporting of a randomised controlled trial (RCT), enabling readers to understand a trial's design, conduct, analysis and interpretation, and to assess the validity of its results.
Cytology	The study of cells; their origin, structure, and function.
D	
Dual Stained Cytology (DS)	Immunostaining for the simultaneous detection of the coexpression of p16 and Ki-67.
DNA (Deoxyribonucleic Acid)	The genetic material of all cellular organisms. Genetic material is replicated during the S-Phase of the cell cycle.
GP	General Practitioner.
H	
High-Grade Squamous Intraepithelial Lesion (HSIL)	In the Australian context, usually used to refer to a cytological category predictive of the presence of a high grade precancerous lesion, (histological CIN 2 or CIN 3).<br>pHSIL (possible HSIL) in the Australian Modified Bethesda system is broadly equivalent to ASC-H; whereas dHSIL (definite HSIL) is broadly equivalent to HSIL in the standard US Bethesda System of cytological classification.

Human Research Ethics Committee	A group of experts who review clinical trial protocols to make sure that the rights of the patient are protected.
Human Papillomavirus (HPV)	Common name for a group of related viruses of which around 40-50 have a predilection for genital (and sometimes oro-pharyngeal) epithelium. These genital types usually result in subclinical (invisible to the naked eye) infection, but some can also cause genital warts. Most HPV infection is transient but persistent infection with high-risk or oncogenic types may lead to the development of high grade cervical abnormalities and invasive cervical cancer.
HPV vaccination	Prevents infection with certain types of human papillomavirus associated with the development of cervical cancer, genital warts, and some less common cancers.
Hysterectomy (total)	Complete surgical removal of the uterus including the cervix.
**I**	
Intraepithelial	Within the layer of cells that form the surface or lining of a part of the body.
Automated Image Read Liquid Based Cytology (Auto LBC)	Computer assisted image analysis for liquid based cytology screening.
**L**	
Low-Grade Squamous Intraepithelial Lesion (LSIL or low grade abnormalities).	In Australian context, usually used to refer to a cytological category predictive of the presence of a low grade precancerous lesion, (histological CIN 1) although some proportion of cytological LSIL is also associated with high grade disease (CIN 2/3).<br><br>pLSIL (possible LSIL) in the Australian Modified Bethesda System is broadly equivalent to ASCUS; whereas dLSIL (definite LSIL) is broadly equivalent to LSIL in the standard US Bethesda System of cytological classification.
Liquid Based Cytology (LBC)	Liquid based cytology (LBC) is a way of preparing cervical samples for examination in the laboratory.
**M**	
Medical Services Advisory Committee (MSAC)	MSAC is an independent scientific advisory committee comprising individuals with expertise in clinical medicine, health economics and consumer matters. They provide advice to the Australian Minister for Health and Ageing on evidence relating to the safety, effectiveness and cost-effectiveness of new medical technologies and procedures.
**N**	
National Cervical Screening Program (NCSP)	A joint program of the Australian, state and territory governments. It aims to reduce morbidity and mortality from cervical cancer, in a cost-effective manner through an organised approach to cervical screening. The program encourages women in the target population to have regular Pap smears.
National Cervical Cytology Coding Sheet	Coding sheet for pathology laboratories reporting cervical cytology.
National Health and Medical Research Council (NHMRC)	Australia's peak body for supporting health and medical research; for developing health advice for the Australian community, health professionals and governments; and for providing advice on ethical behaviour in health care and in the conduct of health and medical

	research.
National Human Papillomavirus Vaccination Program Register (NHVPR)	A registry recording information about HPV vaccine doses administered in Australia.
Oncogenic HPV	Potentially cancer-causing HPV DNA types, pathogenically linked to intraepithelial neoplasia–e.g., uterine cervix, termed CIN.
**P**	
Partial HPV genotyping	Testing for selective strands of HPV DNA.
P16/Ki-67	p16INK4a (p16) and Ki-67 are validated cell cycle regulatory proteins which are a markers of active cell proliferation.
Pap Test	Test developed by George Nicholaus Papanicolaou in the 1940s in which cells are scraped from the cervix and examined for abnormal cells that could indicate cancer.
Positive predictive value (PPV)	Proportion of positive test results that are true positives (such as correct diagnoses).
Practitioner	General Practitioner, specialist or nurse Pap test provider: someone who performs routine cervical screening.
**S**	
SNOMED Systematised Nomenclature of Medicine	Classification system used to report the diagnosis for the cell type of the malignant disease.
Screening to Prevent Cervical Cancer: Guidelines for the Management of Asymptomatic Women with Screen Detected Abnormalities 2005-NHMRC	Guidelines from the National Health and Medical Research Council on the management of women without symptoms who have screen detected cervical abnormalities.
**T**	
Triage	The process of determining the priority of patients' treatments based on the severity of their condition.
**V**	
Verification Bias	In this context, a potential bias in the assessment of the accuracy of a screening test, when diagnostic verification (in this case, using colposcopy) is only performed when a screen-detected abnormality is present; thus in this case there is the possibility that the assessment of screening test accuracy does not take into account disease missed by the screening test.

Classification of risk

Throughout the document we refer to various definitions of levels of risk for women in the population, based on their primary screening results. The table below defines these categories and describes their relationship to the current diagnostic categories for cytology according to the Australian Modified Bethesda System.

Table 1. Risk classification in relation to current National Cervical Screening Program.

Risk Stratification	Cytology Category	Primary HPV Category
**Unsatisfactory**	Unsatisfactory	Unsatisfactory
**Low risk**	Negative	Negative for HPV
**Intermediate risk**	p/d LSIL	Other oncogenic HPV#
**High risk**	p/d HSIL	HPV 16/18

excludes 16/18

2. Summary

This document describes the protocol for a pilot study for the *Compass* trial, a randomised controlled trial (RCT) of primary HPV DNA screening to be conducted in Australia. Women aged 25-64 years, attending for routine cervical (Pap test) screening at participating GP and other medical practices in the state of Victoria will be asked to consent to participate in the 3-arm trial. A liquid-based cytology (LBC) sample will be taken and returned to VCS Pathology at the Victorian Cytology Service (VCS). Samples will be randomised at the laboratory in a 1:2:2 parallel group allocation using a computer-generated automated randomisation scheme with sequential number generation, with randomisation stratification by age group (25-29 years and 30-64 years).

The screening and management algorithms for the three study arms will be as follows: *ARM 1*. 2.5-yearly image read cytology screening with reflex HPV testing of women with low grade cytology (p/d LSIL) (control arm); *ARM 2*. 5-yearly HPV screening with reflex HPV genotyping, referral of HPV16/18 positive women to colposcopy, and cytology testing of intermediate-risk women positive for other oncogenic HPV types; and *ARM 3*. 5 yearly HPV screening with HPV 16/18 genotyping, referral of HPV16/18 positive women to colposcopy, and dual-stained (DS) cytology (with p16/Ki67) testing of intermediate-risk women positive for other oncogenic HPV types. Please refer to Figures 1-3 for full management pathways for each arm. The laboratory reports issued to practitioners will specify the recommended management for women, according to study arm and test results.

A pilot study of 5,000 women (1,000 in the cytology screening and 2,000 in each primary HPV study arm at a 1:2:2 randomisation allocation) will recruit participants in the first year (2013). The pilot study aims are:

(i) To assess participant acceptance of the randomisation process and the use of longer routine screening intervals by quantifying the study recruitment rate for invited participants (overall and by practice);

(ii) To confirm the operational feasibility of laboratory processing procedures by quantifying, for two HPV test technologies, the sample volume requirements for each required testing process and the hands-on and total processing time;

(iii) To assess positivity rates for the primary screening test in each arm in women <30 years and 30+ years, and to perform preliminary cross-sectional analyses to estimate sensitivity and specificity in the baseline screening round for histologically-confirmed Cervical Intraepithelial Neoplasia grade 3 (CIN 3) or invasive cancer in each arm (i.e. CIN 3+); and

(iv) To perform a sub-study to estimate the sensitivity and specificity of dual-stained cytology testing for the detection of CIN 2+ and CIN 3+.

We plan to follow women enrolled in the pilot study for 5 years as outlined in this protocol and their results will be included in the main trial analyses. Once 5,000 women have been recruited to the pilot and their samples tested, the protocol will be reviewed by the investigators in conjunction with the advisory committee and finalised prior to seeking ethics approval for the main trial. If there are any material changes to the protocol with implications for information provided to participants at the time of consent, then we will write to women enrolled in the pilot to inform them of the changes and remind them that they are free to withdraw from the study at any time.

Participating women will be flagged on the Victorian Cervical Cytology Registry (VCCR) and invitation letters will be issued from the registry 3 months prior to the designated time for re-screening. Participant follow-up will also be tracked via the VCCR. Data linkage between the VCCR and the National HPV Vaccination Program Register will eventually be performed to obtain complete screening and vaccination histories for trial participants.

A random subsample of screen-negative and reflex-test negative women in the baseline round will be referred for colposcopy, in order to control for verification bias in cross-sectional analyses of the baseline findings. A local pathologist will perform histopathology for primary patient care. An independent quality control panel comprising three expert pathologists who will not be aware of the study arm or screening or triage test results will conduct a secondary review of the histology to provide rigorous, scientific endpoints. A total of 5% of women in the HPV testing arms will be randomly selected to undergo LBC testing at 2.5 years for safety monitoring purposes. A Data Safety and Monitoring Board will be configured to review these follow-up data.

Following the completion of recruitment for the pilot study, the outcomes will be analysed and participants will be followed-up as part of the main trial. The main trial objectives will be finalised after the pilot study. An *example* formulation of the primary objectives for the main trial (currently proposed) for an anticipated total sample size of 100,000 women is as follows:

(i) To confirm that the cross-sectional sensitivity ratio for the detection of CIN 3+ in the baseline screening round for HPV testing versus cytology is >=1.0 (i.e. non-inferiority of HPV testing for cross-sectional detection of CIN 3+);

(ii) To confirm that the cumulative hazard of developing histologically-confirmed CIN 3+ at 6 years for women who were HPV-negative at baseline is equivalent or less than the cumulative hazard at 3 year follow-up for women who were cytology-negative at baseline, and equivalent or less than the cumulative hazard in the subgroup of these who were also repeat cytology negative at 6 years (i.e. non-inferiority of HPV testing for longitudinal outcomes, based on an expected rate of ~0.51% at 3 years in the cytology arm and an average rate of ~0.27% at 6 years in the HPV test arms);

(iii) To confirm that the ratios for cross-sectional sensitivity and specificity for the detection of CIN 3+ in the baseline screening round for Arm 2 vs. Arm 3 is >=1.0 (i.e. non-inferiority of dual-stained cytology vs. LBC as a triage test in women with other oncogenic type infections after primary HPV screening and direct referral of HPV 16/18 positive women for cross-sectional detection of CIN 3+).

Compass will be conducted in parallel with a major review of the National Cervical Screening Program (NCSP) in Australia. It is anticipated that results from the pilot study, will feed into and inform this review as they become available. The longitudinal outcomes from the pilot study and the main trial will provide information which will inform optimisation of the future national screening program.

3. Introduction

3.1 Current status of cervical screening in Australia

The implementation of the NCSP in Australia has resulted in substantial reductions in cervical cancer incidence and mortality since its inception in 1991.[1;2] The program currently recommends screening with

conventional cytology every 2 years for sexually active women aged 18-20 to 69 years,[3] and achieves high 2-yearly and 3-yearly participation rates.[4] Despite the very considerable success of the screening program, a number of issues have arisen that have prompted consideration of potential changes. The recommended 2-yearly screening interval and relatively wide age range of screened women means that screening is conducted more intensively in Australia than in most developed countries,[5;6] and the National Health and Medical Research Council has recommended a review of the interval.[7] Given the low rates of cervical cancer incidence and mortality in Australia, and scientific support for the maintenance of cytological screening efficacy with a 3-yearly interval and older age of starting screening (at aged 25 years),[8;9;10] consideration is being given to changing these aspects of the organised program. The International Agency for Research on Cancer (IARC) has recommended that for cytological screening, the optimal screening interval is 3 years in women aged 25-49 years, and 5-years in women aged 50-64 years. The IARC recommendations are based on a body of international evidence that suggests that little benefit is gained by screening women more frequently; thus these intervals for screening represent the best balance of harms and benefits of cytological screening [37].

A major driver for future change to the screening program is the implementation of the National HPV Vaccination Program. A universal access school-based vaccination program in females using quadrivalent HPV 16/18/6/11 vaccine commenced in April 2007, with community-based catch-up vaccination to 26 years implemented to end-2009.[11] Vaccines based on HPV16/18 virus-like particles have been shown to be highly effective in preventing persistent HPV infection and the development of precancerous cervical intraepithelial neoplasia (CIN) in females naïve to HPV vaccine types.[12;13] The vaccines may also provide some protection against non-vaccine oncogenic HPV types, although the degree to which cross-protection exists has not generally been quantified to a high level of precision.[14] Because 2-yearly cervical screening continues to be recommended for sexually active women between the ages of 18-70 years, vaccinated and screened cohorts already overlap. The implementation of the National HPV Vaccination Program was performed early in the international context; it has one of the widest reported age ranges for publically funded catch-up vaccination; one of the higher levels of vaccination coverage reported in any country to date (approximately 73% in 12-13 year old girls[11] and ~30% in the older catch-up cohorts aged 20-26 years in 2007)[15] and one of the youngest ages of starting screening.[16] Therefore, Australia is likely to be the first setting in the world in which large numbers of vaccinated women will participate in population-based cervical screening, which will still be necessary because current HPV vaccines provide only partial protection against cervical cancer.

HPV vaccination is expected to reduce the positive predictive value of screening for predicting the future occurrence of precancerous disease by decreasing the number of underlying cytological abnormalities (which could have a de-training effect on laboratory readers) and shifting the distribution of abnormalities towards low grade cytological abnormalities.[17] For this reason, and to allow for the development of consistent screening recommendations for both vaccinated and unvaccinated women, optimising cervical screening in the context of HPV vaccination is likely to require changes to future screening policy. Vaccination has been demonstrated not to affect the clearance of pre-existing HPV infections.[12;18;19] Therefore, women positive for a specific oncogenic HPV type should be considered to be at similar risk of developing a precancerous lesion in the future, irrespective of their prior vaccination status. Thus, primary HPV testing for the presence of any oncogenic type could allow for the simplification of screening recommendations in the post vaccination era. This is an important consideration because the vaccination status of the population targeted for screening will vary over time as more vaccinated cohorts (through the catch-up program) reach screening age.

In 2009, the Medical Services Advisory Committee (MSAC) in Australia reported on evaluations of new cytology technologies, including manually read liquid-based cytology (LBC), automated image read LBC

(AutoLBC), and HPV triage testing for low grade cytological abnormalities.[20;21] At that time MSAC found that the cost-effectiveness ratios associated with use of these technologies were high and appeared unfavourable, but the evaluation also demonstrated that the cost-effectiveness of LBC became more favourable if screening was performed 3-yearly. As a consequence the technologies were rejected for public funding, but it was noted that "If changes to the Australian screening program are considered in the future, and as changes due to vaccination are realised, reassessment of the cost-effectiveness of these technologies, using similar methods, would be warranted as part of any review of screening strategies and technologies."

Primary HPV DNA testing has been reviewed and endorsed as a primary screening method by the International Agency for Research on Cancer.[22] Evidence from overseas clinical studies suggests that the introduction of HPV DNA testing for primary screening would allow a further extension of the screening interval of up to 5-7 years, and in recent years a significant body of evidence has emerged to support this from a number of international randomised controlled trials on primary HPV testing.[23;24;25;26;27;28;29;30;31] The findings of the trials generally suggest that compared to cytology, primary HPV testing has an increased sensitivity for high grade precancerous disease which can result in increased detection of high grade abnormalities in an initial round of screening and consequently reduced rates of disease, including invasive cervical cancer, in follow up screening rounds. Although some trials have used all high grade abnormalities as an endpoint (cervical intraepithelial neoplasia grade 2 or above; CIN 2+), some have also demonstrated lower rates of the immediate cancer precursor or invasive cancer; CIN 3+[32]. A number of longitudinal observational studies have also reported lower rates of CIN 3+ in HPV negative compared to cytology negative women over time. For example, the Joint European Cohort Study found that the cumulative rate of CIN 3+ in HPV DNA-negative women at 6 years after HPV testing was 0.27% (95% CI: 0.12-0.45%); compared to the rate at 3 years for women who were cytology negative at baseline, which was 0.51% (95% CI: 0.23-0.77).[33]

A number of technologies for HPV testing are available. The technology for which the most clinical evidence is available is Qiagen's Hybrid Capture 2 (HC2) (QIAGEN N.V, Netherlands). However, a new generation of alternative HPV testing platforms are also emerging, including the COBAS 4800 technology (Roche Molecular Systems Inc, Pleasanton, CA) and the Abbott RealTime PCR (Abbott Molecular Inc, Des Plaines, IL). Many HPV test platforms now have the ability to perform partial genotyping (i.e. stratifying outputs for HPV positive samples with respect to whether the highest risk HPV types [16/18 and potentially 45] are present, versus other oncogenic types).[34;35] Various options for the management of HPV positive women have been proposed including cytological triage, partial genotyping, and the use of dual-stained cytology for the overexpression of molecular markers such as, for example, cyclin-dependent kinase inhibitor $p16^{INK4a}$ (p16) and Ki-67. Partial genotyping, potentially in conjunction with the other approaches, appears a highly promising strategy which could allow high volume clinical testing with further risk stratification, allowing the differential (more aggressive) management of women exposed to the HPV types most often found in cervical cancer or of those who are positive for progression markers. For example, in the US based ATHENA trial of the COBAS 4800 technology, among HPV-positive women, detection of HPV16, HPV18, or both had better sensitivity (182/252 [72·2%]; p<0·0001) and similar PPV (182/1314 [13·9%]; p=0·70) for detection of CIN 3 or worse than Atypical Squamous Cells of Uncertain Significance (ASCUS) worse cytology alone.[36]

The emerging body of international evidence on the efficacy of primary HPV screening, the advent of HPV vaccination, and the longstanding debate about the frequent screening interval in Australia have been some of the factors prompting a major review of the NCSP. This review, the "Renewal" of the NCSP, was announced in November 2011. Among other issues it will consider the use of new technologies (manually and image-read LBC and primary HPV screening); increasing the interval of

screening; and changing the age of starting screening to 25 years. The first and second phases of Renewal (systematic review of the literature and economic evaluation of screening options) are due to be conducted in 2012-2013, in parallel with the initiation of the Compass trial.[38]

If the Renewal process determines that any change is appropriate to the NCSP, and if it also concludes that cytologically-based screening should be retained, then because of the structure and terms of reference of this currently ongoing evaluation, this will necessarily imply a transition to IARC-recommended age ranges and intervals.[38] Thus the comparator arm for cytological practice in the Compass trial has been designed to reflect this potential outcome of Renewal and also to reflect a 'best current practice' approach to cytology-based screening. The comparator arm will therefore involve the most current technology for cytological screening (image-read LBC with HPV triage of low grade cytology (p/dLSIL)) combined with the internationally recommended 3-yearly cytological screening interval.

This choice of comparator for the Compass trial contrasts with the existing screening recommendations in Australia, which are now being reconsidered (i.e. screening with a 2-yearly interval with conventional cytology in women aged 18-20 to 69 years). However, the choice of comparator for Compass is designed to take account of the international evidence and better balance the harms and benefits of cytological screening. By using the IARC intervals and age range the number of screening tests and the downstream sequelae of colposcopic evaluation and treatment of CIN (including some level of 'over-treatment' of lesions that would have regressed naturally) will be reduced. This is expected to benefit women and potentially also their children, via a potential reduction in the risk of CIN treatment-associated obstetric complications.[40] For example, it has recently been estimated that increasing the screening starting age to 25 years in Australia would reduce the number of preterm deliveries and low birth weight events by up to 142 and 136, respectively, in the lifetime of a cohort of 100,000 unvaccinated women; by 65 and 63 in a cohort of vaccinated women; and by 129 and 124 in unvaccinated women who were in a birth cohort who were offered vaccination as 12-13 year olds (with the baseline risks modified due to the effects of herd immunity in this group).[39.] Because cervical screening in women <25 years is of limited effectiveness in preventing invasive cervical cancer[41], raising the screening start age is predicted to have only a minimal effect on rates of invasive cervical cancer, even in unvaccinated women.

The Compass pilot study will recruit women in 2013, and recruitment for the main trial is expected to commence in 2014. Cross sectional results for the pilot and preliminary results from the main study are anticipated to be available in 2013-2014, within the timeframe of the Renewal process. Should the Renewal result in the introduction of primary HPV screening (one of the options under consideration), it is anticipated that the trial sites will act as sentinel sites for rollout of HPV primary screening in Australia. Should the Renewal result in the introduction of LBC based screening, again, it is anticipated that the trial sites will act as sentinel sites for rollout of LBC screening, and will also enable further collection of data to enable future (re-)consideration of primary HPV screening.

4. Objectives

4.1 Pilot Study Objectives

The objectives of the Compass Pilot Study are as follows:

(i) Recruitment Rate. To assess participant acceptance of the randomisation process and the use of longer routine screening intervals by quantifying the overall study recruitment rate for invited participants; and to quantify the recruitment rate for each participating practitioner.

(ii) Operational feasibility. To confirm the operational feasibility of laboratory processing procedures for each of two alternative HPV test platforms (Qiagen HC2 and Roche COBAS 4800). This will be done by quantifying, for each technology, the sample volume requirements for each required testing process and the hands-on and total processing time (which will be used to estimate the laboratory processing costs). The main objectives will be to assess whether there is a difference in the percentage of cases in which the available sample is insufficient to complete all tests described in the protocol, requiring sample recollection of the sample, so called "unsatisfactory" screening episodes and of more than 3% in total processing time between the two technologies.

(iii) Test positivity rates. To assess positivity rates for the primary screening test in each arm in women <30 years and 30+ years, and to perform preliminary cross-sectional analyses to estimate the sensitivity and specificity in the baseline screening round for histologically-confirmed CIN 3 in each arm.

(iv) Dual-stained (DS) cytology assessment. To estimate the sensitivity and specificity of DS testing, in women positive for HPV.

4.2 Proposed Main Trial Objectives

A *proposed* formulation of the primary objectives for the main trial for an anticipated total sample size of 100,000 women is as follows:

(i) To confirm that the cross-sectional sensitivity ratio for the detection of CIN 3+ in the baseline screening round for HPV testing versus cytology is >=1.0 (i.e. non-inferiority of HPV testing for cross-sectional detection of CIN 3+);

(ii) To confirm that the cumulative hazard of developing histologically-confirmed CIN 3+ at 5 years for women who were HPV-negative at baseline is equivalent or less than the cumulative hazard at 2.5 year follow-up for women who were cytology-negative at baseline, and equivalent or less than the cumulative hazard in the subgroup of these who were also repeat cytology negative at 5 years (i.e. non-inferiority of HPV testing for longitudinal outcomes, based on an expected rate of ~0.51% at 2.5 years in the cytology arm and an average rate of ~0.27% at 6 years in the HPV test arms);

(iii) To confirm that the ratios for cross-sectional sensitivity and specificity for the detection of CIN 3+ in the baseline screening round for Arm 2 vs. Arm 3 is >=1.0 (i.e. non-inferiority of dual-stained cytology vs. LBC as a triage test in women with other oncogenic type infections after

primary HPV screening and direct referral of HPV 16/18 positive women for cross-sectional detection of CIN 3+).

Prior to ethical review for the main trial, these proposed main trial objectives will be reviewed, presented and discussed with the study Scientific Advisory Committee, and finalised in light of results from the pilot.

5. Methods

5.1 Qualitative work

Prior to ethical review, a consumer review of all participant documentation will be carried out in a population sample of eligible women, coordinated through the Cancer Council NSW. It will involve sending out a questionnaire in a sample of approximately 30-50 women aged 25-64 years and asking questions about the readability and understanding of the Participant Information and Informed Consent form.

To identify acceptability, supportive factors and barriers to consent, additional focus groups will be held with participating practitioners early in the initial recruitment phase. Should any concerns with the recruitment rates be experienced, secondary focus groups will be held upon the completion of the pilot study. Qualitative information elicited from practitioners will be used to identify themes raised as barriers to the acceptability of the consent process, and additional information materials may be developed for practitioners and patients.

5.2 Recruitment rates (Objective 1)

The targeted recruitment rate for the pilot study is 50-70% (corresponding to a range of 7,143 to 10,000 women invited in order to obtain 5,000 women recruited). However, the expected rate within that range is 65%. The expected recruitment rate is based on a number of data sources including the following:

- Experience from a trial of Chlamydia screening (the trial is known as 'CIRIS') conducted in Victoria via general practitioners, which recruited 1,116 of 1,698 invited participants for a recruitment rate of 66% (95% CI: 63-68%). Therefore if the Compass pilot study achieved comparable rates of recruitment, between 7,885 and 7,355 women would need to be invited to participate in order to have 5,000 women enrolled.

- International experience from a trial of primary HPV screening conducted in Canada, a somewhat comparable screening environment (CCCaST). This trial recruited 9,667 of 14,482 invited participants for a recruitment rate of 67% (95% CI: 66-68%). Therefore if the Compass pilot study achieved comparable rates of recruitment, between 7,579 and 7,406 women would need to be invited to participate in order to have 5,000 women enrolled.

5.2.1 Analysis Plan

Recruitment rates will be calculated using information from VCS Pathology. VCS Pathology records information by individual practitioner which can be aggregated to obtain overall information for a participating practice.

The recruitment rate (by practitioner and overall) will be calculated as follows - the numerator will be women from whom a consent is received, from whom an LBC sample has been taken, who are verified at receipt of sample at VCS Pathology as being eligible, and who are then randomised to one of the study arms. The denominator will be the number of women from whom VCS Pathology receives a cervical screening sample who fulfil the trial eligibility criteria, who are either enrolled in the trial as above or who are not enrolled in the trial.

The target recruitment rate for participating individual practitioners will be 50% or greater. Recruitment rates for each practitioner will be calculated in blocks of 20 Compass-eligible women (as defined above). If individual practitioners have a lower level of recruitment during the progress of the pilot study a review of the recruitment processes will be held to identify particular issues or barriers. No formal ‘stop’ rule will be specified, however, if overall recruitment in the pilot is less than 50%, study materials will be reviewed and additional focus groups may be held with practitioners and patients.

The final pilot study recruitment rates will be reported by practitioner and age (<30 and 30+ years), as well as overall. Using data obtained from VCS Pathology a comparison of socio-demographic characteristics for participants versus non participants will be reported by age group, Local Government Area (LGA), number of screening tests in the last 5 years and, after linkage, vaccination status (as recorded by the NHVPR).

5.3 Operational Feasibility (Objective 2)

We will calculate for each technology the percentage of screening episodes in which we were unable to undertake all tests as described in the protocol. In these cases, a summary report of “Unsatisfactory: insufficient sample for testing” will be issued and a recommendation for a repeat sample collection will be made.

Sample volumes will be assessed pre- and post-testing in 100 samples for each technology. We will generate a descriptive report of the overall mean pre-test sample volume, total mean sample volume required (post-pre-test volume) required for each different test performed and the average number and type of tests performed on the sample following a positive and negative primary screen test for each study arm. Volumes will be estimated by weighing samples before and after removal of fluid for each testing step.

A “Time and Motion” study will be used to measure work-load and resources for the cytology arm and two HPV testing arms, including the number and type of technicians involved in each processing step. As part of the Time and Motion study, several measures will be reported, including total laboratory processing times (TLT), total sample processing times (TPT) and hands-on processing times (HOT).

A timesheet and resources log will be recorded by a study research assistant (RA) or other qualified staff member for each process observation.

5.3.1 Analysis Plan

Total laboratory processing start time will be defined as time and date a sample is received and logged at specimen reception to the time when the final report is entered into the laboratory electronic database. Total sample processing time (TPT) will be defined as the time from specimen load to HPV result being available on the manufacturer's output. For hands-on time (HOT), an RA will observe and record the start and stop of the hands-on processing times. A minimum of three sample runs/batches per test (up to ~90 test samples per run) will be conducted. The average results across the three runs will be taken to derive averages of the three key measures for each technology.

The time and motion study will be conducted after suitable run in and lab verification has been performed for each technology. In the pilot study it is unlikely that the full batch capacity will be utilised, due to the need to report in a timely fashion. Therefore adjustments will be made to allow for components of the processing time that are dependent on the number of individual samples versus automated components of the processing time that are independent of the number of samples being processed.

Descriptive times will be calculated for all pathways in the flow diagrams of Figures 2 and 3.

For DS, HOT for preparation of the stain using an immunostainer will be quantified. Reading/scanning with microscopy time will also be quantified.

Similar processes will be followed for quantifying HOT and TOT in the cytology arm.

5.4 Test positivity rates (Objective 3)

Test positivity rates will be reported by age (overall, <30 and 30+ years) in the cytology arm and both HPV arms. We will also use data from screen-negative women referred to colposcopy to estimate the proportion of true negative and false negative results in each arm, and will thus estimate the cross-sectional sensitivity and specificity of each screening test.

5.4.1 Analysis Plan

- Using participant's Round One screening test results, obtained from VCS Pathology, we will estimate the test positivity rates by age (overall, <30 and 30+) for the primary screening test in each arm (Cytology: HPV: HPV).
- Using test result data from screen negative women that were referred for colposcopy as part of the verification colposcopy arm we will estimate the cross-sectional sensitivity and specificity of each primary screening test (Cytology: HPV: HPV). These data will be collected at the time of colposcopy, by attending specialists, and recorded by the VCCR.

5.5 Dual Stained (DS) Cytology Sub-study (Objective 4)

We will perform a sub-study to estimate the sensitivity and specificity of DS for the detection of CIN 2+ and CIN 3+ in women positive for HPV.

In the pilot study, all women positive for HPV in the primary HPV arms (irrespective of HPV type) will be considered for inclusion in the sub-study, as will all women undergoing verification colposcopy. DS will be

performed if enough sample is available following the completion of the laboratory tests required for management in the trial. For each HPV-positive woman for whom DS testing is performed, post-hoc age matching will be performed with HPV-negative women who were selected for referral to colposcopy.

The study will aim to assess, for HPV16, HPV18 and for other oncogenic types, the sensitivity and specificity of the HPV DS combination for detecting histologically confirmed CIN 2+ and CIN 3+. In addition, the sensitivity and specificity of DS from within the pool of HPV positive women will be calculated (for each HPV type).

Although it is not possible to estimate precisely the proportion of women that will be HPV positive in the trial (since available data are associated with some statistical uncertainty and the final distribution of age groups recruited in the pilot study and the precise effect of vaccination on HPV rates in younger women has not been characterised), it is likely that the rate of HPV positive results in the trial overall will be of the order of 5-7%. This would suggest that of the 4,000 women in the HPV primary testing arm in the pilot study, approximately 200 will be HPV positive; these women will have DS testing in the pilot study. In addition, a further 200 age and practice-matched HPV-negative women in the verification arm will have DS testing.

5.5.1 Analysis Plan

Sensitivity and specificity of the Dual-Staining test will be calculated as follows:

		1.1.1.1 **Disease**	
		Present	Absent
**1.1.1.2 Test outcome**	Positive test	True positive (A)	False Positive (B)
	Negative test	False Negative (C)	True Negative (D)

$$Sensitivity = \frac{A}{A + C}$$

$$Specificity = \frac{B}{B + D}$$

5.6 Linear Array Sub-Study

The Linear Array Sub-study will involve retesting the liquid based cytology sample material with the Linear Array HPV Genotyping test. This test individually identifies 37 HPV genotypes and will be used for research purposes only. The rationale for re-testing is that we have identified an apparent fall in HPV prevalence due to vaccination (which is expected), and wish to confirm this via comparison with a prior pre-vaccination study (Garland et al., 2011, Human Papillomavirus prevalence among indigenous and non-indigenous Australian Women prior to a national HPV vaccination program-WHINURS Study), which used the Linear Array technology. By re-testing the samples, we will be able to estimate the extent to which technology differences can explain the observed fall in HPV prevalence. The linear array will give us a benchmark of the differences between a research-based test and the diagnostic testing which is currently used as a population based screening tool. We will also be able to validate the clinical HPV technology used in Compass for ongoing monitoring of HPV vaccine impact via Compass and via the new renewed cervical screening program which will use HPV testing (to be introduced 2017).

The samples will be re-tested at VCS Pathology using a Linear Array system; and a small subset (<200) will be re-tested again at the laboratories of Roche Molecular Systems (RMS) in Pleasanton, USA as part of our verification process, required when introducing new technology into the laboratory.

The subset of samples sent to RMS, will be de-identified. They will be allocated a unique number that will enable each sample to have its RMS and VCS Pathology result compared. No other information will be provided. As these tests are for research purposes only we will not let participants know the outcome of the results and it will not influence or affect the ongoing management of participants in the Compass trial.

We have already informed participants via the Participant Information Sheet that participation in the trial means that the remainder of their material may be used for future research.

6. Pilot Study Design

6.1 Design

Compass is a three armed randomised controlled trial of image read cytology screening versus primary HPV DNA testing in Australian women aged 25-64. The trial will be initiated in the setting of the regular NCSP, with recruitment conducted through Primary Health Care Centres (PHCC) (GP, family planning and sexual health clinics). The trial will have a parallel group design, with randomisation to one of three arms at a 1:2:2 allocation ratio.

6.2 Sample size

The pilot study will involve recruiting 5,000 women aged 25-64 years. In early 2012, the VCS Pathology Liaison Physicians provided a comprehensive list of all general practices with close links to VCS. Pathology. From the list, 6 eligible practices were identified and have agreed to participate in the trial. The participating practices are: Ballarat Group Practice (Victoria Street, Sturt Street, Howitt Street); Clifton Hill Medical Practice; Collins Street Medical Centre; Melbourne Sexual Health; Midtown Medical Clinic and the Brooke Street Medical Centre. These clinics report approximately 6,300 Pap tests from age eligible women per year.

The initially proposed sample size of 100,000 for the Main Trial is based on the expected rate of cumulative accumulation of serious precancerous disease or invasive cancer (CIN 3+) in screen-negative women in each of the study arms, using international data to inform these expected rates. In the Pilot Study, in order to obtain an accurate estimate of the recruitment rate, the acceptability of the increase in screening interval and different tests, the operational feasibility in the lab, and to establish benchmark population parameters for use for the Main Trial, we have estimated that a sample size of 5000 women aged 25-64 is required (5% of the sample size of the Main Trial).

Estimates of the expected per-centre recruitment rate in each age strata was performed for Pilot study Objective (i) and sample size calculations were performed for each of the Objectives (ii)-(iv) as follows:

(i) <u>Recruitment Rate</u>.

Using data on the number of cervical screening samples sent to VCS Pathology in 2012 by each participating clinic, we estimated the expected number of women aged 25 – 29 and 30 – 64 years that would be enrolled in the pilot study if the recruitment rates were 65% and 50% overall, and if these recruitment rates were also sustained by each individual clinic. (As detailed in Section 5.2, the target recruitment rate for participating individual practitioners will be 50% or greater, and if overall recruitment in the pilot is less than 50%, study materials will be reviewed and additional focus groups may be held with practitioners and patients during the course of the pilot).

Assuming a 65% recruitment rate, we obtain the following estimates of the number of recruited participants at each centre.

	25-29	30-64	Total
**BALLARAT GROUP PRACTICE**	130	1100	1230
**MELBOURNE SEXUAL HEALTH CENTRE**	386	509	895
**COLLINS STREET MEDICAL CENTRE**	116	756	872
**CLIFTON HILL MEDICAL GROUP**	102	773	875
**MIDTOWN MEDICAL CLINIC**	146	797	943
**BROOKE STREET MEDICAL CENTRE**	30	452	482
**Total**	907	4383	5290

Assuming a 50% recruitment rate, we obtain the following estimates of the number of recruited participants at each centre.

	25-29	30-64	Total
**BALLARAT GROUP PRACTICE**	100	846	946
**MELBOURNE SEXUAL HEALTH CENTRE**	297	391	688
**COLLINS STREET MEDICAL CENTRE**	89	581	670
**CLIFTON HILL MEDICAL GROUP**	78	594	672
**MIDTOWN MEDICAL CLINIC**	112	613	725
**BROOKE STREET MEDICAL CENTRE**	23	348	371
**Total**	697	3371	4068

(ii) Operational feasibility

The main outcome measure with a bearing on sample size will be the TPT per sample. Based on a prior study (as yet unpublished, Wheeler et al), an average mean TPT per sample for COBAS 4800 of 3.15 min is expected. The table below shows the required sample size to detect a difference at the required

level (with the difference being either positive or negative), at a power of 0.8 and a significance level of 0.05, under two sets of assumptions about the standard deviation (SD) in TPT for each test. As shown in the table, a sample size of 90 samples (average over 3 batches) will allow the detection of differences in TPT of the order of 3%, even assuming a larger intra-test variation (SD=0.2 min).

Cobas 4800 TPT per sample	% difference between technologies	Sample size required, if SD=0.1 min	Sample size required, if SD=0.2 min
3.15	3%	18	71
3.15	4%	10	40
3.15	5%	7	26
3.15	6%	5	18
3.15	7%	4	13
3.15	8%	3	10
3.15	9%	2	8
3.15	10%	2	7

(iii) Test positivity rates, sensitivity and specificity

Given an estimate of the true test positivity rate for LBC and HPV testing in the population, we can calculate the maximum 95% confidence interval widths that can be expected given that we have 1,000 women in the LBC Cytology arm (Study Arm 1) (200 <30, 800 30+) and 2000 in each HPV arm (Study Arms 2 and 3) (400 <30, 1,600 30+), taking into account the expected size of the final groups in each age stratum. These age strata estimates were obtained from the data on the number of cervical screening samples sent to the VCS Pathology in 2012 by each of the participating clinics.

	**Primary screening test**				
	**LBC**		**HPV**		
	**Age group (test positivity estimate)**		**Age group (test positivity estimate)**		
	**<30 (4%)**	**30+ (1.5%)**	**<30 (Unvaccinated) (13%)**	**<30** (Assuming vaccination approximately halves positivity rate **(7%)**	**30+ (7%)**
**95% CI widths**	0.05	0.02	0.07	0.05	0.03

Therefore, at the proposed study sample size, we expect to be able to estimate the test positivity rate for LBC and HPV in each age stratum with a precision of better than +/-3% and +/-4% respectively.

For a given expected sensitivity, we can calculate the number of participants with the disease required to achieve a given precision when we calculate our sensitivity estimate. Similarly, for a given expected specificity we can do the same calculation. The following tables give the required number of participants for different 95% confidence interval widths. Furthermore, we estimate the precision of the sensitivity and specificity estimates that will be obtained for the detection of histologically confirmed CIN 3+ for the expected recruited number of women (for the assessment of sensitivity, in the first instance we will assume that the rates of disease identified in screen-negative women in the verification colposcopy group are representative of all screen-negative women in each study arm).

	Expected HSIL Sensitivity	Expected HSIL Specificity
**HPV Estimate 1**	**83.9% (71.7% - 92.4%)**	**76.7% (74.5% - 81.3%)**

95% Confidence Interval Width	Number of CIN3+ women required	Number of women required in HPV study arm (0.5% CIN3+)	Number of <CIN3 women required
**0.2**	52<br>(27 - 78)	10400<br>(5400 – 15600)	69<br>(59- 73)
**0.1**	208<br>(108 - 312)	41600<br>(21600 – 62400)	275<br>(234 - 292)
**0.05**	831<br>(432 - 1248)		1099<br>(935 - 1168)
**0.02**	5190<br>(2698- 7796)		6866<br>(5841 - 7299)

The following table shows the maximum 95% confidence interval widths that can be expected if we assume that the true HPV sensitivity and specificity are as above, and that we have 2,000 in each HPV arm.

	Sensitivity	Specificity
**95% CI widths**	0.46<br>(0.33 – 0.56)	0.037<br>(0.034 – 0.038)

Therefore, at the proposed study sample size, we expect to be able to estimate the sensitivity of HPV testing with a precision of better than +/-23% and the specificity with a precision of better than +/-2%.

	Expected HSIL Sensitivity	Expected HSIL Specificity
HPV Estimate 2	92.7% (82.4% -98.0%)	70.3% (64.7% - 75.5%)

95% Confidence Interval Width	Number of CIN3+ women required	Number of women required (0.5% CIN3+)	Number of <CIN3 women required
**0.2**	26<br>(8 - 56)	5200<br>(1600 – 11200)	81<br>(72 - 88)
**0.1**	104<br>(31 - 233)	20800<br>(6200 – 46600)	321 (285 - 351)
**0.05**	416<br>(121 - 892)		1284 (1137 - 1404)
**0.02**	2600<br>(753 - 5572		8021 (7106 - 8774)

The following table shows the maximum 95% confidence interval widths that can be expected if we assume that the true HPV sensitivity and specificity are as above, and that we have 2,000 in each HPV arm.

	Sensitivity	Specificity
**95% CI widths**	0.32<br>(0.17 – 0.47)	0.04<br>(0.038 – 0.042)

Therefore, at the proposed study sample size, we expect to be able to estimate the sensitivity of HPV testing with a precision of better than +/-16% and the specificity with a precision of better than +/-2%.

	**Expected HSIL Sensitivity**		**Expected HSIL Specificity**
**LBC**	**57%**<br>**(46% - 67%)**	**72%**<br>**(Australian estimate Creighton et al 2010)**	**97% (94% - 99%)**

**95% Confidence Interval Width**	**Number of CIN3+ women required**		**Number of women required (0.5% CIN3+)**		**Number of <CIN3 women required**
**0.2**	95<br>(85 - 96)	78	19000<br>(17000 – 19200)	15600	12<br>(4 - 22)
**0.1**	377<br>(340 - 382)	310	75400<br>(68000 – 76400)	6200	45<br>(16 - 87)
**0.05**	1507<br>(1360 - 1527)	1240			179<br>(61 - 347)
**0.02**	9416<br>(8494 - 9543)	7745			1118<br>(381 - 2167)

The following table shows the maximum 95% confidence interval widths that can be expected if we assume that the true LBC sensitivity and specificity are as above, and that we have 1,000 in the LBC arm.

	Sensitivity		Specificity
**95% CI widths**	0.87<br>(0.82 – 0.87)	0.79	0.021<br>(0.012 – 0.03)

Therefore, at the proposed study sample size, we expect to be able to estimate the sensitivity of cytology testing with a precision of better than +/-40% and the specificity with a precision of better than +/-1%.

(iv) Dual-stained (DS) cytology assessment.

The following sample size calculation tables show the number of participants with/without the disease (CIN 2+ or CIN 3+) required to obtain different 95% confidence interval widths when calculating the CIN 2+ and CIN 3+ sensitivity and specificity of DS in HPV+ women. For the sensitivity estimates, the tables also show the total number of HPV+ women needed in order to have the required number of women with CIN 2+/CIN 3+ (assuming 20% CIN 2+ and 13.7% CIN 3+ in HPV+ women respectively, as observed in the Netherlands POBASCAM trial in Round One 43

95% Confidence Interval Width	Number of CIN2+ women required	Number of HPV+ women required (20% CIN2+ in HPV+ women)	Number of <CIN2 women required
**0.2**	97	485	97
**0.1**	385	1925	385
**0.05**	1537	7685	1537
**0.02**	9604	48020	9604

95% Confidence Interval Width	Number of CIN3+ women required	Number of HPV+ women required (13.7% CIN3+ in HPV+ women)	Number of <CIN3 women required
**0.2**	97	709	97
**0.1**	385	2811	385
**0.05**	1537	11219	1537
**0.02**	9604	70103	9604

The following tables show the maximum 95% confidence interval widths that can be expected if we assume that the true DS CIN 2+ and CIN 3+ sensitivity and specificity are 90%, and that we have 2,000 in each HPV arm.

	Sensitivity (CIN2+)	Specificity(CIN2+)
**95% CI widths**	0.16	0.08

	Sensitivity (CIN3+)	Specificity(CIN3+)
**95% CI widths**	0.19	0.08

Therefore, at the proposed study sample size, we expect to be able to estimate the CIN 2+ and CIN 3+ sensitivity of DS testing in HPV positive women with a precision of better than +/-8% and +/10 respectively, and the specificity with a precision of better than +/-4%.

6.3 Participants

Eligible women will be aged 25-64, presenting for routine cervical screening at participating PHCC in Victoria, Australia. At a later point, the trial may also be extended to include Family Planning practices throughout Australia. Women with a history of cytological or histological abnormalities or who have been treated for high grade CIN in the past will be eligible as will those presenting for an early repeat screening test following unsatisfactory cytology reports, providing that the current visit is for the purpose of routine screening.

Only women who are willing and able to provide informed consent will participate in the trial.

<u>Inclusion Criteria</u>

- Australian female resident of Victoria aged 25-64 years.
- Attending for routine cervical screening at participating Primary Health Care Clinics (PHCC) or sexual health clinics in Victoria (or follow-up of prior unsatisfactory smear for routine screening).

<u>Exclusion Criteria</u>

- Previous total hysterectomy (uterus and cervix).
- The presence of symptoms for which cervical cancer must be excluded.
- Currently undergoing treatment for cervical pre-cancer, or cancer.
- Attending for follow-up of a prior cervical abnormality, including repeated “test of cure” procedures in which the woman has not yet been discharged back to routine screening.
- Known pregnancy.

6.4 Interventions

The screening and management pathways for the three study arms are shown in Figures 1-3 and are defined as follows:

Control - Arm 1: two and a half-yearly image read cytology screening with reflex HPV triage testing for low grade smears;

Intervention - Arm 2: five-yearly HPV screening with types 16/18 (+/-45 in pilot study) genotyping and cytology triage of intermediate risk women with other oncogenic HPV infection; and

Intervention - Arm 3: five-yearly HPV screening with types 16/18 (+/-45 in pilot study) genotyping and dual-stained (DS) cytology (with p16/Ki67) triage of intermediate risk women with other oncogenic HPV infection.

Figure 1. Management Processes for Study Arm 1.

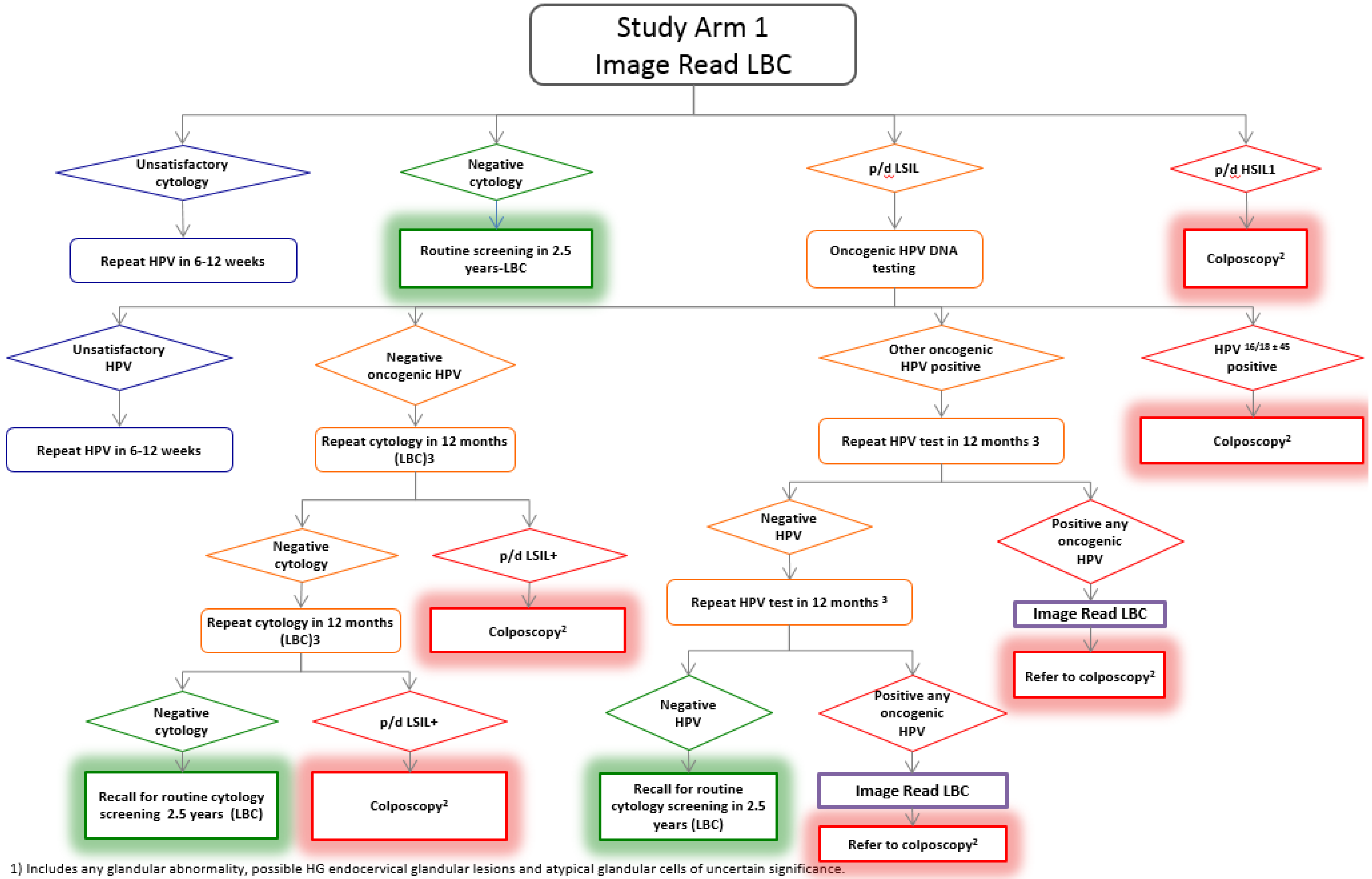

1) Includes any glandular abnormality, possible HG endocervical glandular lesions and atypical glandular cells of uncertain significance.
2) If results at colposcopy are negative/CIN 1/HPV, women require two negative follow-up tests at 12 months and 24 months, using index test, before returning to routine screening (return to original study arm). If CIN 2+/AIS: treatment and follow-up according to the NCSP guidelines, for AIS this will be annual co-testing (HPV and LBC) indefinitely. Refer to the National Pathology Accreditation Advisory Council (NPAAC) draft protocol. Colposcopy unsatisfactory: managed by the individual specialist, informed by the NCSP guidelines (see detailed management flow charts).
3) Unless the National Cervical Screening renewal states otherwise.
4) Pregnant women will be excluded, however if a woman becomes pregnant during the course of the trial and presents with an incident abnormality, she will be managed according to trial protocol . Any subsequent colposcopy will be managed as per the 2005 NCSP guidelines-for pregnant women.

Version 2_07Oct2016

Figure 2. Management Processes for Study Arm 2.

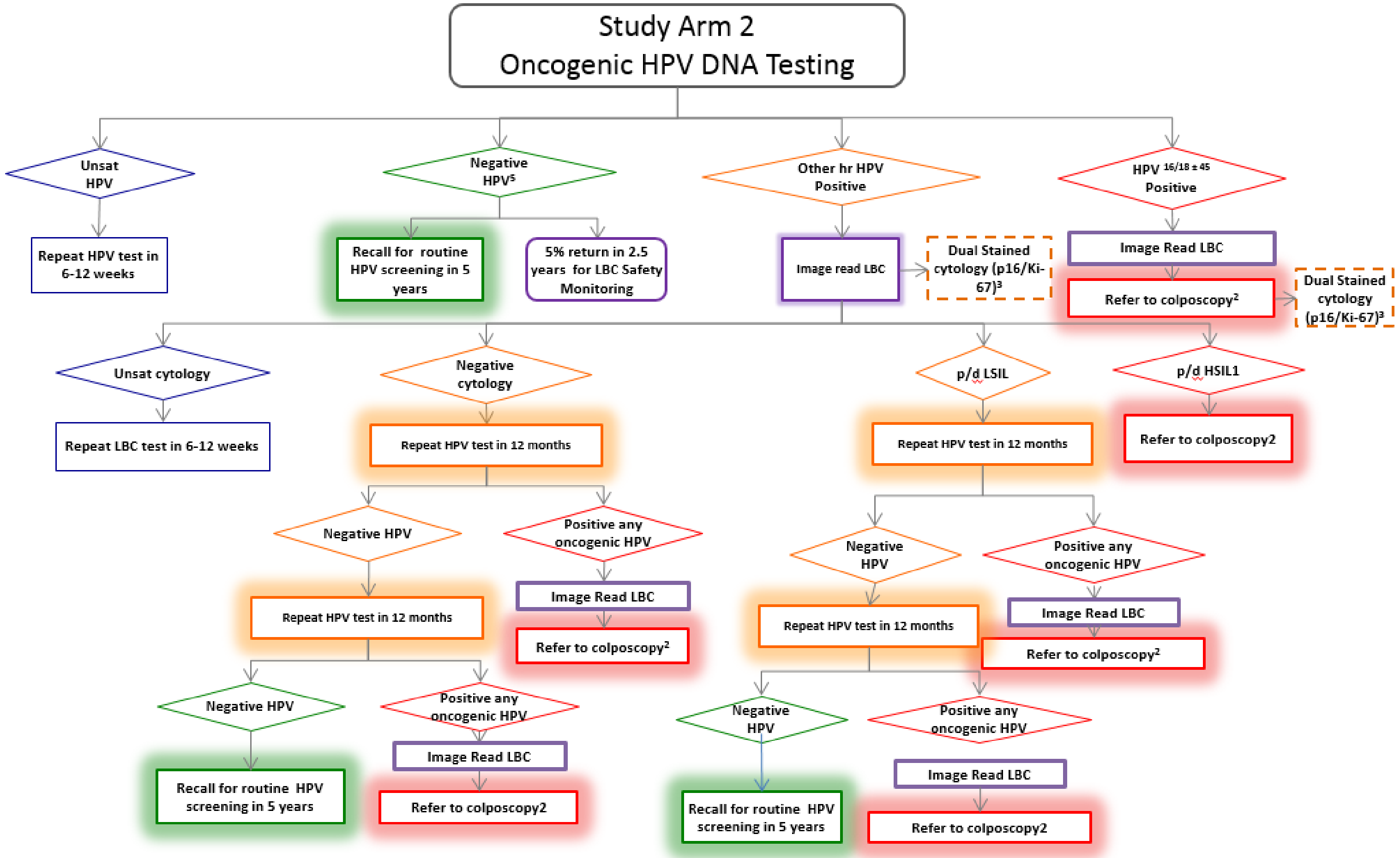

1) Includes any glandular abnormality, possible HG endocervical glandular lesions and atypical cells of uncertain significance.

2)If results at colposcopy are negative/CIN1/HPV, women require two negative follow-up tests at 12 months and 24 months, using index test, before returning to routine screening (return to original study arm). If CIN 2+/AIS: treatment and follow-up according to the NCSP guidelines, , for AIS this will be annual co-testing (HPV and LBC) indefinitely. Refer to the National Pathology Accreditation Advisory Council (NPAAC) draft protocol. (see detailed management flow charts). Colposcopy unsatisfactory: managed by the individual specialist, informed by the NCSP guidelines (see detailed management flow charts).

3)Concealed results: not for management of women

4)Sample of HPV negative will have DS in the Pilot; concealed results: not for the management of women.

5)Pregnant women will be excluded, however if a woman becomes pregnant during the course of the trial and presents with an incident abnormality, she will be managed according to trial protocol . Any subsequent colposcopy will be managed as per the NCSP guidelines-for pregnant women.

Version 2_07Oct2016

Figure 3. Management Processes for Study Arm 3.

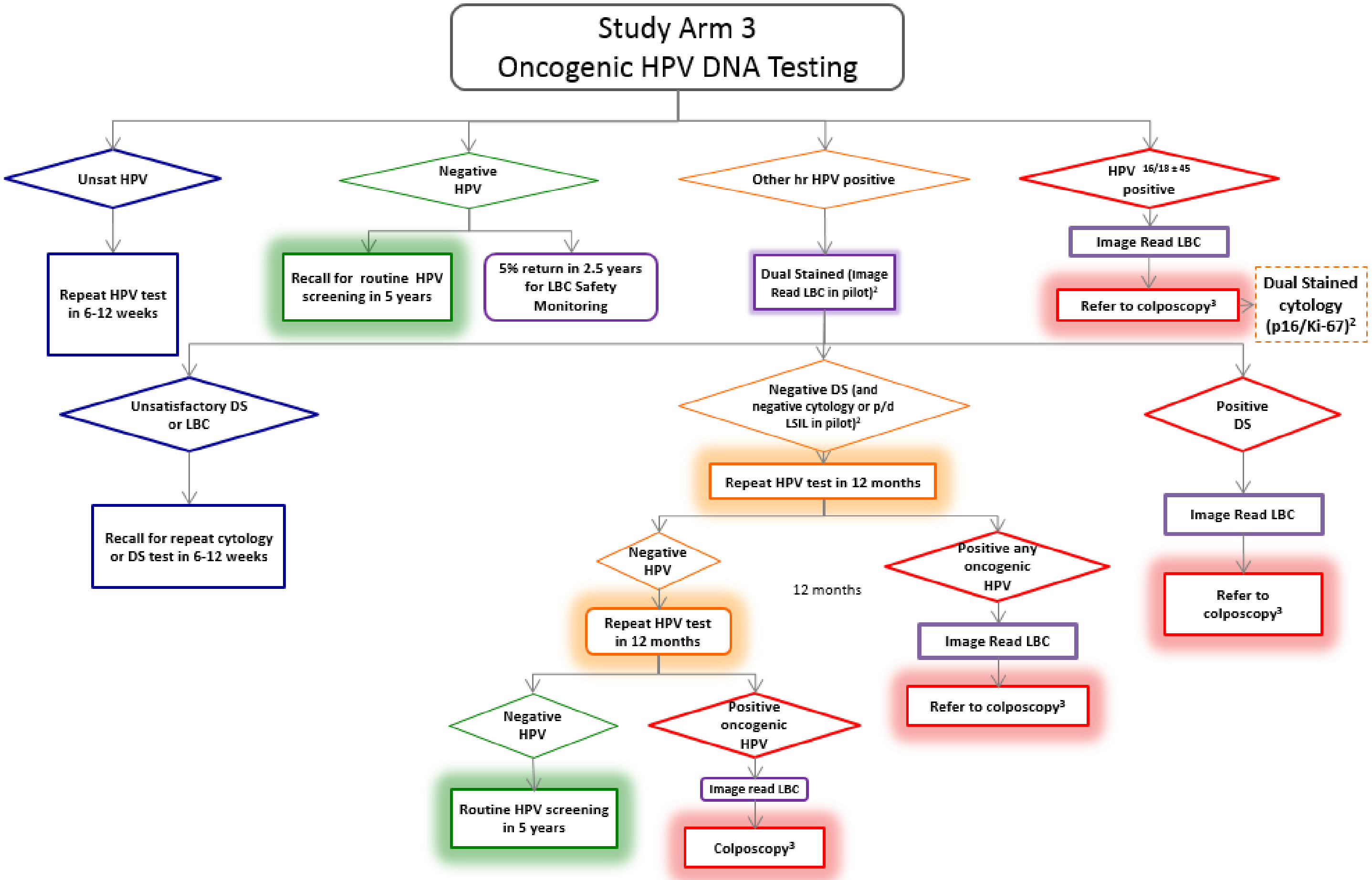

1) Includes any glandular abnormality, possible HG endocervical glandular lesions and atypical cells of uncertain significance.
2)Sample of HPV negative will have dual stained cytology performed in Pilot; concealed results: not for the management of women.
3)If results at colposcopy are negative /CIN 1/HPV, women require two negative follow-up tests at 12 months and 24 months, using index test, before returning to routine screening (return to original study arm). If CIN 2+/AIS: treatment and follow-up according to the NCSP guidelines, , for AIS this will be annual co-testing (HPV and LBC) indefinitely. Refer to the National Pathology Accreditation Advisory Council (NPAAC) draft protocol. (see detailed management flow charts). Colposcopy unsatisfactory: managed by individual specialist, informed by the NCSP guidelines.
4)Pregnant women will be excluded, however if a woman becomes pregnant during the course of the trial and presents with an incident abnormality, she will be managed according to trial protocol . Any subsequent colposcopy will be managed as per the NCSP guidelines-for pregnant women.

Version 2_07Oct2016

6.5 Screening and triage test technologies

The following technologies have been selected for use in the Pilot study. A review of technologies will be performed at the conclusion of the pilot study and before the main trial commences.

Specimen Collection Technology: Samples in the trial will be collected using a liquid-based sample medium. The technology to be used will be PreserveCyt/ThinPrep (Hologic Inc, Bedford MA). Included on the Australian Register of Therapeutic Goods (ARTG)

Image read LBC technology: The Hologic Thinprep Imaging (TPI) System will be used for image read analysis. Included on the ARTG

HPV DNA testing technology: In the pilot study, primary HPV DNA testing will be performed using two technologies. These will be:

(i) Rapid throughput Hybrid Capture (HC2), Qiagen, (QIAGEN, N.V., Netherlands). For Qiagen technology HPV positive samples will be re-run using a probeset for HPV 16/18/45. Included on the ARTG

(ii) COBAS 4800 HPV Test (Roche Molecular Systems Inc., Pleasanton,CA). Roche COBAS system provides integrated genotyping for HPV 16/18. Included on the ARTG

    (i) Cobas 4800 System, Sample Preparation Kit: c4800 SMPL PREP
    (ii) Cobas 4800 System, Amplification/Detection Kit: c4800 HPV AMP/DET
    (iii) Cobas 4800 System, Preparation Kit: c4800 LIQ CYT
    (iv) Cobas 4800 System, Wash Buffer Kit: c4800 WB

Dual Stained cytology (DS). Analysis of dual stained cytology for p16/Ki-67 markers in the trial will involve use of the CINtec® PLUS kit (VENTANA MEDICAL SYSTEMS, INC., , AZ). The DS in-vitro diagnostic medical device is included on the ARTG.

Follow up of women will be performed using:

(i) COBAS 4800 HPV Test (Roche Molecular Systems Inc., Pleasanton,CA). Roche COBAS system provides integrated genotyping for HPV 16/18. Included on the ARTG

    (i) Cobas 4800 System, Sample Preparation Kit: c4800 SMPL PREP
    (ii) Cobas 4800 System, Amplification/Detection Kit: c4800 HPV AMP/DET
    (iii) Cobas 4800 System, Preparation Kit: c4800 LIQ CYT
    (iv) Cobas 4800 System, Wash Buffer Kit: c4800 WB

6.6 Biobanking

If women consent to participation in the study, residual sample left after all tests specified in the protocol will be stored for later retrospective testing for research purposes with one or more alternative test technologies. Residual samples in LBC vials will be stored for a minimum of 1-3 months according to usual laboratory practices (in case repeat testing is required for any reason) and may be retained longer,

or at a later time, cell pellets may be spun down and frozen as whole cells. This will allow for later assessment of DNA, RNA or protein biomarkers. Samples will be labeled with a unique ID and stored in a secure freezer located at VCS Pathology or at another appropriate site for which appropriate contractual and governance arrangements for storage are in place. This will create a biobank resource, comprising population-based samples for which linkage to the results of histopathology analysis, screening test history, trial outcomes and other data will be performed. This resource will allow for future assessment of differences between true and false positive rates between different HPV test technologies, partial typing systems, and progression markers. The parallel testing using alternate technologies will not be used to manage women and will be performed retrospectively. The results of these tests will not be made available to women or their doctors (i.e. the results of testing will be 'concealed'). It is anticipated that separate funding will eventually be sought for testing Biobank samples. Ethical approval for the biobanking process in the Pilot will be sought as part of the process of seeking approval for the Pilot.

7. Recruitment and Clinical Management

7.1 Coordinating Centres and Study Investigators

The coordinating centres will be the Cancer Research Division at the Cancer Council NSW (CCNSW), Sydney, and the Victorian Cytology Service (VCS Inc.) Inc. which includes VCS Pathology, the Victorian Cervical Cytology Registry (VCCR) and the National HPV Program Register (NHVPR), Melbourne, Australia. Recruitment and laboratory work will be coordinated and performed at VCS Pathology, and ethics approvals, protocol development and data analysis will be coordinated and conducted at CCNSW.

VCS Pathology is an accredited, government funded pathology laboratory, reporting around 280,000 cervical cytology tests per year. This represents approximately half the cervical smears taken annually in Victoria. VCS Pathology places a strong emphasis on practitioner liaison. VCS Pathology has the capability to perform image analysis cytology and dual-stained cytology for molecular progression marker analysis.

The Cancer Screening Group at CCNSW, which will coordinate Compass activities at CCNSW, is involved in a number of other projects in cervical cancer epidemiology including the New Zealand Women and HPV study, studies of cervical cancer patterns of care in Australia and Canada, and cervical screening behaviour in migrant women in NSW, Australia. It is also the provider of the Independent Monitoring Reports for the New Zealand NCSP.

The investigative team will involve a number of individuals from VCS Inc., CCNSW and the University of Sydney's Clinical Trials Centre, in addition to a number of other key individuals with specific expertise.

7.2 Informed consent procedures

Practitioners will approach patients during regular cervical screening visits, confirm eligibility and seek consent from women.

Prior to performing the cervical screening examination, the practitioner will assess patient eligibility and, where appropriate, seek consent for participation in the study. The practitioner will be provided with "tear-off" pads of study information sheets to provide to participants (Attachment 1). The consent form will be incorporated at the bottom of the usual pathology request form to VCS Pathology (Attachment 2).

Women who provide consent will have a cervical smear collected using a ThinPrep PreservCyt vial. The LBC sample vial will be labelled with the woman's name and date of birth, placed in a sample bag, along with the consent form, and returned to a centralised processing laboratory at the VCS Pathology.

The information sheet provided to practitioners and a flowchart depicting the recruitment processes are provided in Attachments 3 and 4, respectively.

7.3 Randomisation procedures

Upon receipt and logging of the sample at the VCS Pathology, individual subject allocation to one of the study arms will be performed using a computer-generated randomisation sequence in a 1:2:2 ratio; stratified by age at recruitment (<30 years; 30+ years).

The randomisation schedule and process will be the responsibility of the NHMRC Clinical Trials Centre which has extensive experience in developing and managing the randomisation procedure for clinical trials. Neither the participant nor the practitioner will be aware of subject allocation at the time of the cervical screening visit and LBC sampling. Allocation will not be concealed in the laboratory (for reasons of practicality).

7.4 Laboratory Processes

All cytology and HPV DNA testing will be performed by trained pathologists, scientists and laboratory technicians at VCS Pathology. Molecular tests and preparation of cytology samples will be performed according to the relevant manufacturer's recommendations. Cytology examinations will be reported according to standard internal procedures. Any unsatisfactory test result will be repeated, according to manufacturer's instructions. In study arm 1, pre-aliquots will be taken before LBC reading for reflex HPV testing, in order to prevent any potential contamination of the sample for HPV testing. This practice will be reviewed at the end of the pilot study.

Histology results will be reported according to standard internal procedures by NATA-accredited laboratories in Victoria.

7.5 VCS Pathology Report Processes

The laboratory report issued to the practitioner, from VCS Pathology, will specify which arm of the trial the woman has been randomised to, and whether the woman has been randomised to primary screening with cytology or HPV. The report will give the appropriate management recommendation given the randomisation allocation and test results (according to the flowcharts in Figures 1-3).

In the lower section of the laboratory report there will be a standard test result template for the practitioner to forward to the woman. This will describe the recommended follow-up for the woman. A purse-sized reminder card will be included with the laboratory test result. This card will have the date of next test and a reminder to the practitioner that an LBC sample needs to be collected. As per routine practice, it will be the responsibility of the referring practitioner to forward the test result slip to participating women.

7.6 Verification colposcopy

A proportion of all women in all arms will be randomly selected, at the time of initial study arm allocation, to be invited for baseline verification colposcopy referral. The purpose of verification colposcopy is to estimate the true sensitivity and specificity of the screening and triage tests used in the study. Verification

colposcopy will assist in the statistical correction of potential verification bias; in which only screen (and potential screen-and-triage) positive women are referred for diagnostic evaluation. Verification colposcopy, therefore, implies that a proportion of screen-negative, and screen-positive, triage-negative women are referred to colposcopy to confirm that no disease is actually present.

We will randomly invite 10% of screen-negative and 10% of screen-positive, triage-negative (or triage-low grade), women in the pilot study, with the aim of carrying out colposcopy in 5% of women overall (assuming a 50% attendance rate for those invited) (see Figures 1-3 for more detail). The proportion invited will be reviewed following the pilot based on the observed attendance rate, and the underlying abnormality rate in those attending.

Some women selected at the time of initial study arm allocation for verification colposcopy will be referred to colposcopy in the baseline round in any case, on the basis that their tests indicate that they are higher risk. These women will not receive a separate invitation for verification colposcopy and will be managed through routine colposcopy referral processes.

For the group of women selected to be invited to verification colposcopy, the report sent by the laboratory to the practitioner will include the test result and information that the woman has been selected for verification colposcopy for study purposes. A letter will be sent to the woman, confirming her test results and advising that she has been selected to be invited for verification colposcopy. The letter will further advise the woman that a member of the Compass study team (colposcopy nurse or research assistant) will be contacting her to make the arrangements for the colposcopy visit. This letter will be accompanied by a colposcopy information brochure to assist her in deciding whether she will accept the invitation for colposcopy. A second reminder letter inviting women for verification colposcopy will be sent to women, 2 months after reporting of their screening test.

Should a woman decline invitation for verification colposcopy, or does not attend her scheduled appointment, she will return to routine follow-up, according to the arm to which she was originally randomised. She will be followed up as per protocol by the VCCR and receive a screening reminder letter 3 months before she is due for her next cervical screening visit.

Verification colposcopies will be performed by medical staff from the Dysplasia Clinic of the Royal Women's Hospital, Melbourne. Colposcopists will indicate whether the view of the transformation zone is satisfactory, and will only biopsy areas of the cervix that give rise to clinical concern (as per usual clinical practice). It is anticipated that most women undergoing verification colposcopy will not require biopsy. A sample for cytology testing will not be taken unless there is clinical indication to do so. Any biopsies taken in this context will be sent to VCS Pathology for reporting.

There will be no cost to participants for colposcopy and indicated biopsy for these women. Women will be reimbursed $50, at the time of clinic attendance, for travel, parking and other incidental costs.

7.7 Histopathology

For women referred for diagnostic evaluation, histology analysis will be performed as is routinely done by the pathology laboratory used by the colposcopist. As per normal clinical processes, the pathologist providing the original report will be aware of the findings of the screening and triage tests and other relevant clinical information. For histology performed after verification colposcopy, VCS Pathology will

provide the original report on the samples. All clinical management will be based on the results of routine clinical analysis.

A second histopathology analysis will be performed by an independent quality control (QC) panel comprising three non-local expert pathologists who will not be aware of the study arm, the referral pathway to histology, or screening or triage test results. QC review will be performed on all biopsies taken on women who are in the trial (i.e. verification or clinical biopsies). The QC panel review will be performed in a blinded fashion and the QC pathologists will not be aware of the source of the slides. Histopathology slides reported by other labs will be requested by the investigators to be sent to VCS Pathology for the purposes of coordinating the independent review and then returned.

For women in whom the QC histopathology analysis indicates a previously undiagnosed CIN 2/3+ lesion, their primary practitioner will be made aware of the QC diagnosis. If the woman has not been referred for further evaluation or for treatment of high grade cervical precancerous disease since the biopsy in question was originally taken, the letter to the practitioner will recommend that further investigation is conducted and that treatment of a confirmed high grade lesion proceeds according to existing NHMRC guidelines. In general terms, Compass participants who are treated for CIN 2+ disease will have post-treatment follow-up according to NHMRC guidelines for HPV as “test of cure”, and at the point of treatment will be considered to have completed trial follow-up. Following completion of test of cure these women should return to the routine screening as recommended. Although this group of women will be censored from the main trial analysis from the time of treatment, routine follow-up through the VCCR will continue, and the data will be collated as part of the trial, for safety monitoring purposes and supplementary analyses.

7.8 Safety monitoring-HPV Screening (Arms 2 &3)

A proportion of screen-negative women in the HPV arms will be randomly selected at baseline for safety monitoring with LBC at 2.5 years. This selection process will be conducted in parallel to the selection of women for verification colposcopy to ensure that a different group of women are selected for safety monitoring.

The projected proportion required for the analysis of safety at the 3 year point is 7% of HPV negative women, based on the ability to detect an equal or greater cumulative risk of CIN 3+ in the HPV negative women compared to the cytology negative women at 3 years. However some women are also expected to attend for screening early while others will not attend. Taking all of this into account, 10% will be invited at 3 years to allow for non-attendance. An analysis of a proportion of women attending for safety monitoring in the pilot cohort will be undertaken. If necessary the proportion of women invited for safety monitoring will be adjusted in the main cohort to take account of non-attendance for safety monitoring.

At the point of randomisation to the safety monitoring arm women will be notified via the lab report that they should have a repeat LBC test at 2.5 years. Invitation letters to re-attend screening will be issued to participants at appropriate times (three months prior to the scheduled re-screening interval). Reminder letters (or email/text) will be issued to participants who do not attend within 3 months and a second reminder letter will be sent at 6 months. Follow-up of any abnormalities detected in safety monitoring will be according to study arm 1 (Figure 1).

7.9 Opting-out of the trial

Participants will be informed at the time of consent that they will have the right to opt-out of the trial at any point. Participants will be able to opt-out of the trial by either calling VCS Pathology, the VCCR, the CCNSW (Study Hotline), the recruiting PHCC or the relevant Human Research Ethics Committee (HREC). Instructions to the women on the participant information sheet will provide the study hotline as the primary suggested mechanism to opt out. Once a woman chooses to opt out of the trial, her details on VCS Pathology database (CIS) will be updated to reflect this and her Compass study flag will be changed to an opt out flag. A similar opt-out of trial flag will be marked at the VCCR. These women will subsequently be followed up consistent will National Cervical Screening Policy at the time.

7.10 Transition from Pilot to Main Trial

Women enrolled as part of the pilot will be followed up for a period of 5 years after recruitment. We plan to manage these women in accordance with the pilot protocol as described in this document and their results will be included in the main trial analyses.

However, the pilot protocol will be reviewed by the investigators, in conjunction with the advisory committee, to ensure that it remains appropriate for the main trial. This will take place following recruitment of 5,000 women in the pilot, laboratory testing of their first screening round samples and assessment of outcomes against the pilot trial objectives as outlined in section 4 above.

The finalised main trial protocol and a new NEAF will be submitted for ethics review and, subject to approval by the HREC, the women enrolled in the pilot will be deemed to be participants in the main trial. At this point the pilot study will be closed and final report will be submitted to the approving Ethics Committees.

If there are any material changes to the protocol that have implications for information provided to participants at the time of consent, then we will write to women enrolled in the pilot to inform them of the changes and remind them that they are free to withdraw from the study at any time.

These arrangements are described in the participant information sheet and consent form. Women may elect not to continue into the main trial. We will advise any woman withdrawing from the trial that she should resume participation in the National Cervical Screening Programme.

If the main trial does not proceed for funding or other reasons, we will continue to follow women enrolled in the pilot, for 5years, as described in the protocol.

7.11 Future contact with Participants to enable further research

At the time of consent in the medical practice, women agree that they may be contacted in the future to complete a questionnaire. As one example, a random selection of participants from each study arm and representing each main management pathway in the trial may be contacted to participate in a longitudinal study of health state utilities, or quality-of life aspects related to their screening experience. These women

will be sent a short, self-administered questionnaire. The protocol and related participant materials for any future sub-study will be submitted for review and approval by an NHMRC approved HREC**.**

7.12 Use of trial data for cost-effectiveness or simulation modelling.

Analysis of trial data will be used to inform and update existing epidemiologic and cost-effectiveness models at CCNSW, in order to perform an modelled prediction of the lifetime outcomes associated with each screening and management strategy and also to assess outcomes and cost-effectiveness of a range of variations to the study protocol. Such models may also be used in a range of other epidemiologic and economic evaluations. The data used in these modelled evaluations will be de-identified.

The data used for modelling will include the results of the 'Time and Motion' study, costing data, test positive and negative rates, compliance rates and all other trial results which will be used to inform future modelled analysis, performed as part of NHMRC grants and other research projects undertaken by co-Principal investigator, Prof Karen Canfell's research group at Cancer Council NSW.

7.13 Victorian Cervical Cytology Registry (VCCR) Follow-up

The VCCR routinely follows up cervical abnormalities through reporting of cervical cytology and histology; questionnaires to doctors and Pap test providers; phone calls to doctors; and letters and registered letters to women. The pilot will build upon these established procedures with additional contact by mobile phone and emails for women who cannot be reached through the usual follow-up processes. In addition, reminders for future screening tests will be sent by the VCCR prior to the due date for the next test, rather than after the test is due as is current practice, to ensure that the screening interval is as close as possible to that recommended in each study arm. If the study is informed that a woman has changed doctors, information about the study will be sent to her new doctor prior to the date of her next screening test.

8. Study Monitoring

Compass will be conducted in compliance with the approved scientific protocol and in line with the National Statement on Ethical Conduct in Human Research, NHMRC 2007. No deviation from the protocol will be executed without the prior review and approval of the lead HREC. Any unanticipated necessary deviation from protocol will be immediately reported to the leading HREC according to its standard policies and procedures.

Additionally, researchers will monitor the progress and conduct of the trial through regular reports on recruitment numbers from the VCCR, bi-monthly visits to recruitment sites, reviewing adverse event reports, independent study monitoring and the Independent Data Safety and Monitoring Committee (IDSMC).

8.1 Identifying, Recording and Managing Adverse Events

During the study, the investigators, practitioners and other site staff will be responsible for detecting and documenting events meeting the criteria and definition of an adverse event (AE) or serious adverse event (SAE).

It will be the responsibility of all study personnel to record an AE/SAE when it occurs. Study personnel include investigators, study coordinators, participating medical practitioners, Victorian Cytology Service staff and CCNSW staff. Participating Medical Practitioners will use the Compass Adverse Event Form (Appendix 3-Exec-F-2) to report any adverse event. Personnel internal to the Study will use the form in Appendix 2.

Adverse Event, Serious Adverse Events (as defined below) shall be handled and reported according to VCS's internal procedures, and in compliance with any applicable national and international laws, regulations, and guidelines. VCS shall report all Adverse Events in a timely correct manner to local authorities according to national laws, regulations and guidelines.

8.1.1 Definition of Adverse Events (AE) and Serious Adverse Events (SAE)

An Adverse Event (AE) is any untoward medical occurrence (physical, psychological, social or economic), whether mild, moderate or severe, in a trial subject related to medical management, in contrast to complications of disease. Medical management includes all aspects of care, including diagnosis and treatment, failure to diagnose or treat, and the systems and equipment used to deliver care. Adverse events may be preventable or non-preventable.

Further to the above, a Serious Adverse Event (SAE) is any adverse event that:

- results in death
- is life threatening
- results in hospitalisation
- results in disability or incapacity (persistent or significant)

It is recognised that some cases of Invasive Squamous Carcinoma of the Cervix (ISCC), considered an SAE, will occur in all study arm since no screening regime is likely to completely eliminate all cases.

8.1.2 Documenting and Recording AEs or SAEs

When an AE/SAE occurs, the following will be initially documented and recorded in the participant study record as well as in the adverse event reporting log book. The project coordinator will be responsible for updating and managing the adverse event reporting log book.

- Record each adverse event as separate occurrences, i.e. nausea and vomiting must be recorded as two adverse events.
- Document and describe the adverse event(s) using precise and specific terminology.
- Record the start and stop times of the adverse event(s) as exactly as possible.
- Document the severity of the event(s) Mild, Moderate, Severe, Life-Threatening or Fatal.

- Document any treatment/medication given or action taken in relation to the adverse event.
- Record the outcome of the event.

8.1.3 Evaluating AEs and SAEs

Co-Chief Investigator A/Prof Marion Saville will make an assessment of the intensity for each AE or SAE reported during the trial. The intensity and severity of each AE and SAE should be allocated to one of the following categories:

- **Mild**: An event that only causes minimal discomfort, and is easily tolerated by the patient. It does not interfere with daily activities.
- **Moderate**: An event which causes sufficient discomfort to the patient resulting in interference with everyday activities.
- **Severe**: An incapacitating event resulting in prevention of routine everyday activities.

An AE classified as severe should not necessarily be incorrectly classified as an SAE, since these should be independently evaluated. Both AE and SAE can be classified as severe.

8.1.4 Assessment of Causality

The relationship of the AE or SAE to participation in the trial will be assessed by A/Prof Marion Saville and classified as follows:

- **Not related:** there is no apparent causal relationship between participation in the trial and the adverse event.
- **Unlikely**: it is not reasonable to associate participation in the trial with the documented adverse event.
- **Possible**: participation in the trial may have caused the adverse event.
- **Probable**: the adverse event can be reasonably explained by participation in the trial.
- **Definitely:** the adverse event follows reasonable temporal sequence from participation in the trial.

8.1.1 Practitioner Reporting of AE or SAE

In the event of an adverse reaction or event arising from the taking of a cervical cytology sample or the use of the LBC collection kit, Practitioners will be required to complete the Adverse Events Form. This form will be returned it to Victorian Cytology Service, 265 Faraday Street Carlton, VIC 3053, marked to the attention of Associate Professor Marion Saville who upon receipt will manage according to 8.1.2. 'Reporting, Filing and Electronic Recording'.

8.1.2 Reporting, Filing and Electronic Recording

Any AE or SAE will be reported to the project coordinator, within 24 hours using the Compass Adverse event reporting form found in Appendix 2 or the Compass Adverse Events Form provided to Practitioners (Exec-F-2). The immediate report will contain the start and stop time and date of the event, the severity of the event, any action taken, and the outcome of the event. This may be followed by a detailed written report on the event(s) if requested by the IDSMC or HRECs.

All adverse events, including potentially serious adverse event reports, will be reviewed by the co-Chief Investigator, A/Prof Marion Saville. A/Prof Saville will inform the Alfred Health Ethics Committee of any AE or serious SAE within 24 hours, using the form found in Appendix 1. Any SAE will also be reported immediately (within 24 hours) to the RACGP NREEC, Mr Russell Smiley, russell.smiley@racgp.org.au committee.

A/Prof Saville will inform the Chair of the Independent Data Safety Monitoring Committee IDSMC of any SAE within 15 days. A/Prof Saville will also oversee the compilation of a summary report of all other AEs, every 6 months, for the IDSMC. The IDSMC will evaluate the occurrence of SAE;s, including cases of invasive cervical cancer, according to pre specified criteria.

The rates of SAEs and rates of CIN 3+ in each study arm detected in the first screening round, and in follow-up rounds, will be reviewed periodically by the IDSMC. The IDSMC will report their findings to the trial investigators and the investigators will inform the HREC as appropriate and specified by the IDSMC. The investigators will also provide annual summaries of SAE rates to the HREC.

A/Prof Saville will also inform the Roche affiliate: Roche Diagnostics Australia Pty Limited, Attn: Dr. George Koumantakis, Manager, Scientific & Regulatory Affairs, 31 Victoria Avenue, Castle Hill, New South Wales 2154, Australia. Tel: +61-2-9860-2329 of any AE or SAE relating to the use of Roche products.

Any request by the HREC or IDSMC for further details will be addressed immediately. Upon completion and submission of the detailed written report, to relevant parties, the Project Coordinator will create an electronic copy of the report and file accordingly. The participant study record on the VCS database will be updated with details of the report

8.1.3 Reporting to the Therapeutic Goods Administration

A/Prof Marion Saville will report any suspected AE and SAE relating to the CINTec Plus product and Cobas 4800 to the IVD Sponsor: Roche affiliate: Roche Diagnostics Australia Pty Limited, Attn: Dr. George Koumantakis, Manager, Scientific & Regulatory Affairs, 31 Victoria Avenue, Castle Hill, New South Wales 2154, Australia. Tel: +61-2-9860-2329. Dr. Koumantakis will assist in identifying the appropriate reporting to the TGA.

For all suspected AEs and SAEs relating to other IVDs used in the trial, A/Prof Saville will make an assessment and contact the relevant IVD Sponsor’s Regulatory Affairs Manager (or delegate) to discuss reporting to the TGA.

If at any stage A/Prof Saville is not satisfied with the response of an IVD Sponsor with regard to an AE or SAE then a report can be made as an IVD User directly to the TGA. This can be done at the following link: http://reporting.tga.gov.au/mdir/udir03.aspx

8.2 Quality Assurance and Control

Compass data, files and SOPs will be monitored regularly, and reviewed annually, by the project coordinator to ensure the trial procedures comply with the approved Protocol and HREC requirements.

8.2.1 VCS Inc. Operational Quality Assurance and Control

VCS Inc. has well established documented policies and procedures to cover operations in both technical and non-technical areas of VCS Pathology, VCCR and the NHVPR. The Policy on Management of Health and Personal Information and the VCS Inc. Code of Conduct apply across the Service, although individual department procedures may contain additional specific information and instructions where appropriate. The Quality Assurance Committee (QAC) a sub-committee of the board of directors of VCS Inc.meets monthly to review scientific and operational quality assurance activities.

8.2.2 Laboratory Quality Assurance

VCS Pathology employs only appropriately qualified or experienced staff for laboratory roles and also provides formal training for Medical Laboratory Scientists. Training needs are identified at least annually. VCS Pathology is a specialist gynaecological pathology laboratory which fully complies with AS ISO 15189:2009, NATA and NPAAC standards relevant to its scope of activities. VCS Pathology maintains a comprehensive quality system.

8.3 Data Safety and Monitoring Board (IDSMC)

An Independent Data Safety and Monitoring Committee (IDSMC) will be configured to monitor the safety of participants in the study.

All SAEs will be forwarded to the Chair of the Data Safety and Monitoring Committee immediately and reported as described above to the Ethics Committee and Funding body.

The IDSMC will meet every 6 months to review other adverse events. The IDSMC will also review laboratory quality monitoring data for all associated laboratory testing. These reports are regularly generated at VCS Pathology as part of routine Quality Assurance monitoring.

Formal stopping criteria will be developed by the IDSMC and will be based on ensuring non-inferiority of HPV testing compared with LBC for the detection of CIN 3+ as evidenced by the cumulative CIN 3+ rates observed in the control (LBC) and intervention (HPV testing) arms.

8.4 Data Storage and Security

Data will be collected and stored in several different formats over the duration of the trial. Personal information including date of birth, name, address, email and phone will be collected on the consent form at the time of recruitment. Medical records of participants will be accessed via linkage to a number of routinely collected datasets. These include records stored on the Victorian Cervical Cytology Registry, the National HPV Vaccination Register, Victorian Central Cancer Register, the Registry of Births, Deaths and Marriages and the State and Territory Pap Test registers. All HPV and cytology test results will be recorded and stored in the usual methods as per current practice at VCS Pathology and VCCR. The Victorian Cervical Cytology Registry (VCCR) is managed by VCS Inc. and all VCCR staff are employed by VCS Inc. and adhere to confidentiality policies of VCS Inc.

Because the trial will inherently form part of the woman's cervical screening record, individual participant results and personal information, collected as part of the trial, will be held on the databases of the VCS Pathology and VCCR, as would normally be the case for any woman participating in the cervical screening program. In the screening program, all cervical cytology, histology and HPV test results are routinely forwarded to VCCR from the reporting laboratory, along with personal information for the

purposes of reminders and follow-up, unless a woman chooses to opt-off. If a woman does not have a record at VCS Pathology or VCCR a new one will be created and linked with trial data.

8.4.1 Data Transfer, Storage and Management

Participants will be assigned a unique study ID code at the VCS Pathology. Data will be extracted in a de-identified format on a regular basis from VCS Pathology/VCCR and transferred to authorised staff at CCNSW where it will be stored on secure servers located at the Azure data centres in Sydney and Melbourne, and accessed only by authorised study personnel. Project Staff at CCNSW will maintain the study database and perform statistical analysis.

Trial data will be stored at the Azure data centres in Sydney and Melbourne by CCNSW for a period of at least 7 years after the completion of the project and publication of results. This amount of time will allow for adequate interest, discussion and follow up surrounding study data to occur. Results will be reported in a series of papers, reports and presentations in scientific forums. Results will be published in statistical aggregate form so that no individual subjects are identifiable directly or indirectly. Reports or published forms resulting from this study will be owned by their authors who will be members of the research team assigned to this study. The information collected for Compass will be owned by the research team and reports or published forms resulting from this study will be owned by their authors who will be members of the research team assigned to this study.

8.5 Data linkage

Initial screening test results and follow-up data for the Compass trial will be retained as part of routine data processes for the VCCR. Participants will also be followed-up via linkage to a number of other routinely collected datasets. Data linkage will be undertaken with informed consent of participants; only de-identified data will be available to the study investigators involved in the analysis. Data custodian and ethical approval will be sought to link the trial data for each participant to the National HPV Vaccination Program Register to obtain information on vaccination status, doses delivered and timing of vaccination.

9. Governance

The overall governance structure for the Compass trial is depicted in Figure 4.

9.1 Scientific Advisory Committee

The Scientific Advisory Committee will advise the investigator team on issues related to protocol, operations and any other issues brought by the investigators to the Committee. The Scientific Advisory Committee (SAC) will be chaired by Prof. Bruce Armstrong, Professor of Public Health at the University of Sydney. Final responsibility for protocol or operational decisions will be the joint responsibility of the Co-PIs.

The Scientific Advisory Committee had a trial initiation meeting in November 2011 and approved the preliminary pilot and main study proposals. They agreed to meet again at the point where data from the pilot is available and the final main study design will be presented for review.

9.2 Trial Reporting

Reporting of trial results will be according to the CONSORT 2010 statement (or any later versions of CONSORT that are published in the timeframe of the study).

9.3 Trial Registration and Protocol Availability

The trial is registered on the Australia and New Zealand Clinical Trial Registry (http://www.anzctr.org.au), ACTRN12614000714684, established at the NHMRC Clinical Trials Centre, University of Sydney. This registry is recognised by the World Health Organisation International Clinical Trials Registry Platform (WHO ICTRP) as a Primary Registry.

The clinical protocol will be made available for the duration of the study on the websites of VCS Pathology and CCNSW, and information provided to practitioners will include links to these websites.

Following the pilot phase, trial methods will be reviewed for the main study. Any important changes to methods after the main study commences will be documented and justified, and will appear in reports of the trial findings.

9.4 Human Research Ethics Committee approval

The trial will be conducted according to the “National Statement on Ethical Conduct in Human Research”. Ethical approval will be sought from appropriate certified Human Research Ethics Committees. The CCNSW Human Research ethics committee will be notified that Prof Karen Canfell will hold de-identified trial data for analysis at CCNSW Protocol amendments

Once the Protocol has been signed by the investigators, it will not be informally altered. Neither co-chief Investigators will modify the Protocol without the prior consent of the other in writing. Protocol amendments will pass through appropriate steps before being implemented.

In general any change which might theoretically increase the risk of a participant will require an amendment which will necessitate approval from the leading HREC. Additionally any protocol modification that may impact upon the validity of the study, or result in changes to the Informed Consent form or Information Sheet will require an amendment. All amendments will be reviewed and approved by the HREC before changes to the Protocol and study procedures are implemented.

It will be the responsibility of the investigators, or their nominee, to submit amendments to the HREC for review and approval. Changes to Protocol will not be implemented until an approval letter is provided. Completed and signed amendments will be circulated to all signatories to the original protocol.

The original signed copy of any amendment will be kept by the project coordinator along with the original protocol. All original trial documentation, including the consent form and information sheet, will be kept by the investigators for the appropriate retention period as defined by the lead HREC.

9.5 Insurance and Indemnification

The co-chief investigators institutions (Victorian Cytology Service Inc., CCNSW) will maintain such insurances to provide indemnity for their officers, employees and agents from and against all actions, claims, demands, costs or expenses (including legal costs on a solicitor and own client basis) made,

sustained, brought or prosecuted or in any manner based upon, or occasioned by or attributable to, any injury to any person (including death) or loss of or damage to property which arise from, or as a result of, conducting the Compass trial.

Co-Principal Investigators

- A/Prof Marion Saville
- Prof Karen Canfell

Chief Investigators

- Dr Philip Castle
- Dr Jeff Tan
- A/Prof Dorota Gertig (VCS)
- Dr Julia Brotherton (VCS)
- A/Prof Kirsten Howard (Usyd)
- Dr David Wrede (RWH)
- Dr Sally Lord (CTC)

Key Responsibilities

Protocol development, review and revision

Associate Investigators

- Dr. Stella Heley
- Dr. Lara Roeske
- Gillian Phillips
- Dr. Jane Collins
- Sandy Anderson
- Dr. Michael Caruana
- Jessica Darlington-Brown
- Others to be determined

Key Responsibilities

- Give advice on protocol and operational aspects of trial

Quality Assurance Panel

Histopathology

Chair: A/Prof. Annabelle Farnsworth

Key Responsibilities

- Review histopathology slides in a blinded manner

Data Safety Monitoring Board (Chair: Prof. Michael Quinn)

Key Responsibilities

- Regularly review safety data in a blinded manner
- Recommend study termination if pre specified stopping criteria are met
- Make safety or monitoring recommendations as appropriate

Scientific Advisory Committee (Chair: Prof. Bruce Armstrong)

Key Responsibilities

- Advise on study protocol development
- Annual progress meetings (more frequent if required)
- Review pilot and main trial analysis

Victorian Cytology Service

VCS

Key Responsibilities

- Laboratory Management
- GP Recruitment
- Participant recruitment
- Implement linkage to VCCR & NHVPR

Cancer Council NSW

Key Responsibilities

- Lead protocol design
- Data management
- Lead data analysis and write-up

NHMRC Clinical Trials Centre

Key Responsibilities

- Provision of randomisation mechanism
- Contribute to statistical aspects of protocol design

10. References

(1) Australian Institute of Health and Welfare. Cervical Screening in Australia 2006-2007. Cat. No. CAN 43. 2009. Australian Institute of Health and Welfare. Cancer Series Number 47.

(2) Canfell K, Sitas F, Beral V. Cervical cancer in Australia and the United Kingdom: comparison of screening policy and uptake, and cancer incidence and mortality. Med J Aust 2006; 185(9):482-486.

(3) National Health and Medical Research Council. Screening to Prevent Cervical Cancer: Guidelines for the Management of Asymptomatic Women with Screen Detected Abnormalities. Commonwealth of Australia . 9-6-2005. Canberra, Commonwealth of Australia.

(4) Australian Institute of Health and Welfare. Cervical Screening in Australia 2006-2007. Cat. No. CAN 43. 2009. Australian Institute of Health and Welfare. Cancer Series Number 47.

(5) Canfell K, Sitas F, Beral V. Cervical cancer in Australia and the United Kingdom: comparison of screening policy and uptake, and cancer incidence and mortality. Med J Aust 2006; 185(9):482-486.

(6) IARC Working Group on the Evaluation of Cancer. IARC Handbooks of Cancer Prevention Volume 10: Cervix Cancer Screening. 10 ed. Lyon: IARC Press; 2005.

(7) Australia Government Department of Health and Ageing. National Cervical Screening Program Renewal. http://www health gov au/internet/screening/publishing nsf/Content/ncsp-renewal [2012 [cited 2012 May 1]; Available from: URL:http://www.health.gov.au/internet/screening/publishing.nsf/Content/ncsp-renewal

(8) Canfell K, Beral V, Green J, Cameron R, Baker K, Brown A. The agreement between self-reported cervical smear abnormalities and screening programme records. J Med Screen 2006; 13(2):72-75.

(9) IARC. Screening for squamous cervical cancer: duration of low risk after negative results of cervical cytology and its implication for screening policies. BMJ (Clin Res Ed) 1986; 293(6548):659-664.

(10) Sasieni P, Adams J, Cuzick J. Benefit of cervical screening at different ages: evidence from the UK audit of screening histories. Br J Cancer 2003; 89(1):88-93.

(11) Brotherton J, Deeks S, Campbell-Lloyd S, Misrachi A, Passaris I, Peterson K et al. Interim Estimates of Human Papillomavirus Vaccination Coverage in the School-Based Program in Australia. Commun Dis Intell 2008; 32(4):457-461.

(12) Future II Study Group. Quadrivalent vaccine against human papillomavirus to prevent high-grade cervical lesions. N Engl J Med 2007; 356(19):1915-1927.

(13) Paavonen J, Naud P, Salmeron J, Wheeler C, Chow SN, Apter D et al. Efficacy of human papillomavirus (HPV)-16/18 AS04-adjuvanted vaccine against cervical infection and precancer caused by oncogenic HPV types (PATRICIA): final analysis of a double-blind, randomised study in young women. Lancet 2009; 374(9686):301-314.

(14) Herrero R. Human Papillomavirus (HPV) Vaccines: Limited Cross-Protection against Additional HPV Types. J Infect Dis 2009.

(15) Fairley CK, Hocking JS, Gurrin LC, Chen MY, Donovan B, Bradshaw C. Rapid decline in presentations for genital warts after the implementation of a national quadrivalent human papillomavirus vaccination program for young women. Sex Transm Infect 2009; 85(7):499-502.

(16) Dickinson JA. Cervical screening: time to change the policy. Med J Aust 2002; 176(11):547-550.

(17) Castle PE, Solomon D, Saslow D, Schiffman M. Predicting the effect of successful human papillomavirus vaccination on existing cervical cancer prevention programs in the United States. Cancer 2008; 113(10 Suppl):3031-3035.

(18) Paavonen J, Naud P, Salmeron J, Wheeler C, Chow SN, Apter D et al. Efficacy of human papillomavirus (HPV)-16/18 AS04-adjuvanted vaccine against cervical infection and precancer caused by oncogenic HPV types (PATRICIA): final analysis of a double-blind, randomised study in young women. Lancet 2009; 374(9686):301-314.

(19) Hildesheim A, Herrero R, Wacholder S, Rodriguez AC, Solomon D, Bratti MC et al. Effect of human papillomavirus 16/18 L1 viruslike particle vaccine among young women with preexisting infection: a randomized trial. Jama 2007; 298(7):743-753.

(20) Medical Services Advisory Committee. Automation Assisted and Liquid Based Cytology for Cervical Cancer Screening. MSAC reference 1122, Assessment report. http://www.msac.gov.au/internet/msac/publishing.nsf/Content/BAE45713D7D0FDEBCA257817001CB46D/$File/1122_MSAC_Assessment_Report.pdf . 2009. 2-3-2011.
(21) Medical Services Advisory Committee. Human Papillomavirus Triage Test For Women With Possible or Definite Low-Grade Squamous Intraepithelial Lesions. MSAC reference 39, Assessment report. http://www.msac.gov.au/internet/msac/publishing.nsf/Content/8FD1D98FE64C8A2FCA2575AD0082FD8F/$File/39_MSAC_Assessment_Report.pdf . 2009. MSAC reference 39, Assessment report. 2-3-2011.
(22) IARC Working Group on the Evaluation of Cancinogenic Risks to Humans. IARC Mongraphs on the Evaluation of Carcinogenic Risks to Humans volume 90 Human Papillomaviruses. 90 ed. Lyon: IARC; 2007.
(23) Mayrand MH, Duarte-Franco E, Rodrigues I, Walter SD, Hanley J, Ferenczy A et al. Human papillomavirus DNA versus Papanicolaou screening tests for cervical cancer. New England Journal of Medicine 2007; 357(16):1579-1588.
(24) Cuzick J, Clavel C, Petry KU, Meijer CJ, Hoyer H, Ratnam S et al. Overview of the European and North American studies on HPV testing in primary cervical cancer screening. Int J Cancer 2006; 119(5):1095-1101.
(25) Bulkmans NW, Berkhof J, Rozendaal L, van Kemenade FJ, Boeke AJ, Bulk S et al. Human papillomavirus DNA testing for the detection of cervical intraepithelial neoplasia grade 3 and cancer: 5-year follow-up of a randomised controlled implementation trial. Lancet 2007; 370(9601):1764-1772.
(26) Naucler P, Ryd W, Tornberg S, Strand A, Wadell G, Elfgren K et al. Human papillomavirus and Papanicolaou tests to screen for cervical cancer. New England Journal of Medicine 2007; 357(16):1589-1597.
(27) Cuzick J, Szarewski A, Cubie H, Hulman G, Kitchener H, Luesley D et al. Management of women who test positive for high-risk types of human papillomavirus: the HART study. Lancet 2003; 362(9399):1871-1876.
(28) Cuzick J, Arbyn M, Sankaranarayanan R, Tsu V, Ronco G, Mayrand MH et al. Overview of human papillomavirus-based and other novel options for cervical cancer screening in developed and developing countries. Vaccine 2008; 26:Suppl-41.
(29) Ronco G, Giorgi-Rossi P, Carozzi F, Confortini M, Dalla PP, Del MA et al. Efficacy of human papillomavirus testing for the detection of invasive cervical cancers and cervical intraepithelial neoplasia: a randomised controlled trial. Lancet Oncol 2010; 11(3):249-257.
(30) Kitchener HC, Almonte M, Gilham C, Dowie R, Stoykova B, Sargent A et al. ARTISTIC: a randomised trial of human papillomavirus (HPV) testing in primary cervical screening. Health Technol Assess 2009; 13(51):1-iv.
(31) Kitchener HC, Almonte M, Thomson C, Wheeler P, Sargent A, Stoykova B et al. HPV testing in combination with liquid-based cytology in primary cervical screening (ARTISTIC): a randomised controlled trial. Lancet Oncol 2009; 10(7):672-682.
(32) Cuzick J, Szarewski A, Mesher D, Cadman L, Austin J, Perryman K et al. Long-term follow-up of cervical abnormalities among women screened by HPV testing and cytology-Results from the Hammersmith study. International Journal of Cancer 2008; 122(10):2294-2300.
(33) Dillner J, Rebolj M, Birembaut P, Petry KU, Szarewski A, Munk C et al. Long term predictive values of cytology and human papillomavirus testing in cervical cancer screening: joint European cohort study. Bmj 2008; 337:a1754.
(34) Thai H, Rangwala S, Gay T, Keating K, McLeod S, Nazarenko I et al. An HPV 16, 18, and 45 genotyping test based on Hybrid Capture technology. J Clin Virol 2009; 45 Suppl 1:S93-S97.
(35) Tang N, Huang S, Erickson B, Mak WB, Salituro J, Robinson J et al. High-risk HPV detection and concurrent HPV 16 and 18 typing with Abbott RealTime High Risk HPV test. J Clin Virol 2009; 45 Suppl 1:S25-S28.
(36) Castle PE, Stoler MH, Wright TC, Jr., Sharma A, Wright TL, Behrens CM. Performance of carcinogenic human papillomavirus (HPV) testing and HPV16 or HPV18 genotyping for cervical cancer screening of women aged 25 years and older: a subanalysis of the ATHENA study. Lancet Oncol 2011.
(37) Iarc (2005). IARC Handbooks of Cancer Prevention Volume 10: Cervix Cancer Screening. Lyon, IARC Press

(38) MSAC, Application 1276, Final Decision Analytic Protocol to guide the assessment of the National Cervical Screening Program Renewal. September 2012

(39) Canfell. K., 2012, The Impact of Raising the Age of Starting Cervical screening on both Cervical Cancer and Obstetic Complication rates: Example from Australia (IPV Conference Presentation 2012).

(40) Kyrgiou. M., Koliopoulos. G., Martin-Hirsch. P., Arbyn. M., Prendiville. W., Paraskevaidis. E., 2006. Obstetric outcomes after conservative treatment for intraepithelial or early invasive cervical lesions: systematic review and meta-analysis, The Lancet, 367 (9509).

(41) Sasieni. P., Castanon. A., Cuzick. J., 2009, Effectiveness of cervical screening with age: population based case-control study of prospectively recorded data. BMJ, 339: b2968

(42) Rijkaart. D.C., et at 2012, Human papillomavirus testing for the detection of high-grade cervical intraepithelial neoplasia and cancer: final results of the POBASCAM randomised controlled trial. Lance Oncology; 13: 78-88

Signing page

CO-CHIEF INVESTIGATORS

A/Prof KAREN CANFELL

[illegible]

Signature

Date: 14 Feb 2013

A/Prof MARION SAVILLE

[illegible]

Signature

Date: 15 FEB 2013

OFDNIL
