# Supplementary material for: Cervical screening with primary HPV testing or cytology in a population of women in which those aged 33 years or younger had previously been offered HPV vaccination: Results of the Compass pilot randomised trial
Source: PLoS Med. 2017 Sep 19;14(9):e1002388. doi: 10.1371/journal.pmed.1002388 (PMC5604935; doi:10.1371/journal.pmed.1002388)
Supplement: S2 Text — (PDF) [file pmed.1002388.s002.pdf]

Appendix to the manuscript:

Cervical screening with primary HPV testing or cytology in a population of women in which those aged 33 years or younger were offered vaccination: Results of the Compass pilot randomized trial

Karen Canfell,[1,2,3*] Michael Caruana, [1] Val Gebski, [4] Jessica Darlington-Brown MPH, [1] Stella Heley, [5] Julia Brotherton, [5,6] Dorota Gertig, [5,6,] Chloe J. Jennett, [1] Annabelle Farnsworth, [2,7] Jeffrey Tan, [8,9] C. David Wrede, [8,9] Philip E. Castle, [10] Marion Saville, [5,6]

[1]Cancer Research Division, Cancer Council New South Wales, Sydney, New South Wales, Australia

[2]School of Public Health, Sydney Medical School, University of Sydney, Sydney, New South Wales, Australia

[3]Prince of Wales Clinical School, The University of New South Wales, Sydney, New South Wales, Australia

[4]NHMRC Clinical Trials Centre, University of Sydney, Sydney, New South Wales, Australia

[5]Victorian Cytology Service Ltd., Melbourne, Victoria, Australia *(affiliation during time of the work for DG)*

[6]School of Public Health, University of Melbourne, Melbourne, Victoria, Australia

[7]Douglas Hanly Moir Laboratory, Sydney, New South Wales, Australia

[6]Department of Obstetrics and Gynaecology, University of Melbourne, Melbourne, Victoria, Australia

[9]Department of Oncology & Dysplasia, The Royal Women's Hospital, Melbourne, Victoria, Australia

[10]Albert Einstein College of Medicine, Bronx, New York, The United States of America.

Appendix Section S2.

Verification Colposcopy Recruitment Rates and Outcomes

A proportion (16%) of all screen-negative and also all triage-negative women in all arms of the trial were randomly selected and invited for verification colposcopy performed at the Royal Women's Hospital, Melbourne. It was anticipated that verification colposcopy would assist in the statistical correction of potential verification bias in those women not immediately referred to colposcopy.

In practice it was found that attendance rates for invited women for verification colposcopy were low overall (25%) and likely not to be an unbiased representation of all participants. Because of the challenges associated with interpretation the findings were not used for any calculations in this manuscript. Figure S2 shows the proportion attending verification colposcopy.

Three additional CIN2+ cases (one in the LBC screening group and two in the HPV+LBC triage group) were identified via verification colposcopy. Each of these participants were screen positive, but triage negative i.e. they had screening tests with results indicating intermediate risk (and therefore, if not sent for verification colposcopy would have been followed up in 12 months). The primary and triage test results for these three women are shown below.

1) Participant randomised to LBC screening: The screening result was ASCUS with a triage HPV result of OHR. The final histology diagnosis was severe dysplasia/carcinoma in situ +/- HPV: Classified as **CIN 3.**

2) Participant randomised to HPV+LBC triage screening: The screening result was OHR HPV with a triage cytology result of ASCUS. The final histology diagnosis was moderate dysplasia +/- HPV: Classified as **CIN 2**

3) Participant randomised to HPV+LBC triage screening: The screening result was OHR HPV with a triage cytology result of negative. The final histology diagnosis was moderate/severe dysplasia +/- HPV: Classified as **CIN 3.**

It should be noted that for the Compass main trial, verification colposcopy was not performed, on the recommendation of the Scientific Advisory Committee, after consideration of the early data on attendance rates in this pilot experience, and given that in the main trial the primary endpoint is based on longitudinal CIN3+ (rather than cross-sectional CIN2+) and that all women will received HPV exit testing at five years in the main trial.

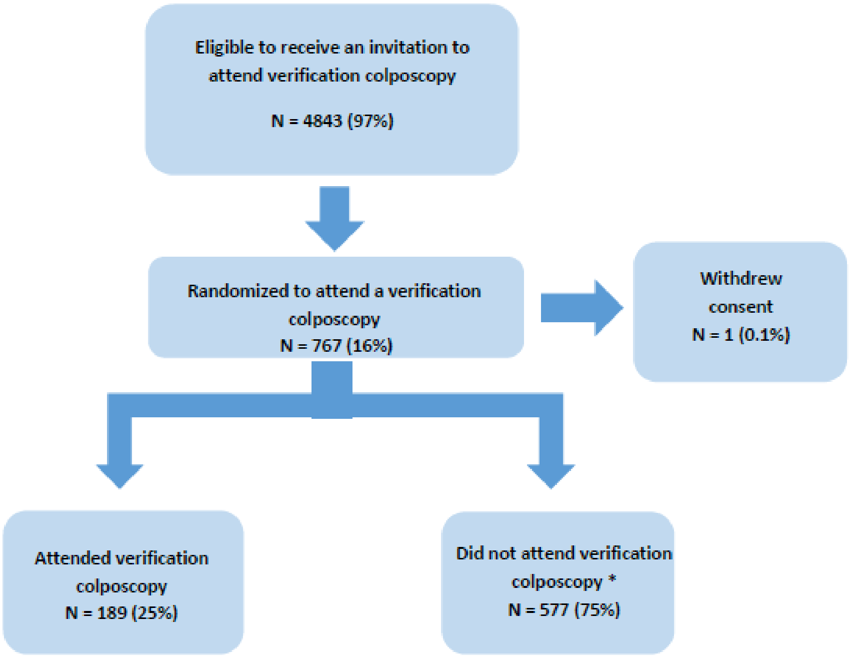

* Unable to be contacted, declined, cancelled their appointment, failed to attend or became pregnant

Fig A. Flowchart showing participation for verification colposcopy
