# Supplementary material for: Cervical screening with primary HPV testing or cytology in a population of women in which those aged 33 years or younger had previously been offered HPV vaccination: Results of the Compass pilot randomised trial
Source: PLoS Med. 2017 Sep 19;14(9):e1002388. doi: 10.1371/journal.pmed.1002388 (PMC5604935; doi:10.1371/journal.pmed.1002388)
Supplement: S3 Text — (PDF) [file pmed.1002388.s003.pdf]

Appendix to the manuscript:

Cervical screening with primary HPV testing or cytology in a population of women in which those aged 33 years or younger were offered vaccination: Results of the Compass pilot randomized trial

Karen Canfell,[1,2,3*] Michael Caruana, [1] Val Gebski, [4] Jessica Darlington-Brown MPH, [1] Stella Heley, [5] Julia Brotherton, [5,6] Dorota Gertig, [5,6,] Chloe J. Jennett, [1] Annabelle Farnsworth, [2,7] Jeffrey Tan, [8,9] C. David Wrede, [8,9] Philip E. Castle, [10] Marion Saville, [5,6]

[1]Cancer Research Division, Cancer Council New South Wales, Sydney, New South Wales, Australia

[2]School of Public Health, Sydney Medical School, University of Sydney, Sydney, New South Wales, Australia

[3]Prince of Wales Clinical School, The University of New South Wales, Sydney, New South Wales, Australia

[4]NHMRC Clinical Trials Centre, University of Sydney, Sydney, New South Wales, Australia

[5]Victorian Cytology Service Ltd., Melbourne, Victoria, Australia *(affiliation during time of the work for DG)*

[6]School of Public Health, University of Melbourne, Melbourne, Victoria, Australia

[7]Douglas Hanly Moir Laboratory, Sydney, New South Wales, Australia

[6]Department of Obstetrics and Gynaecology, University of Melbourne, Melbourne, Victoria, Australia

[9]Department of Oncology & Dysplasia, The Royal Women's Hospital, Melbourne, Victoria, Australia

[10]Albert Einstein College of Medicine, Bronx, New York, The United States of America.

Appendix Section S3.

Findings from the Quality Control re-read for the LBC screening group

Due to the low rates of high grade cytology observed in the LBC screening group, a blinded independent quality control slide re-read was performed at another laboratory routinely performing LBC, Douglas Hanly Moir, Sydney, Australia, after appropriate ethical approval was obtained. All results were reported to the trial IDSMC.

Methods

A Quality Control (QC) re-read of all the Liquid Based Cytology (LBC) sample material from participants randomized to the LBC group was performed. This took place in two stages:

1. The original laboratory (the VCS) carried out a complete manual non-blinded re-read of all LBC samples.
2. This was followed by a blinded QC rescreen performed by Douglass Hanly Moir (DHM) Laboratory, Sydney, Australia. DHM utilised image-based LBC for the rescreen. DHM were informed of the age of the participant, as this is relevant for the correct interpretation of results, however no previous history was provided.

Once the results were received from DHM, VCS then performed HPV testing on those samples where there was any discordance between the original VCS image-read result, the manual VCS result and/or the DHM image-read result. As the women participating in the study had consented to their samples being used for research purposes, they were not notified of the rescreening unless there were changes to their clinical management as a result of a change in test results. This is the standard of care and the process used outside of the research setting

following cytology rescreening in Australia. Ethical approval for the rescreening of samples was received from Alfred Hospital Ethics committee (EC00315).

Results

Table S3.1 shows the findings for the VCS re-read in relation to the original reading. The (weighted) percentage of agreement between the VCS image-read and manually-read LBC results was 98.5%; the weighted kappa-statistic measure of agreement was estimated to be 0.87, representing almost perfect agreement beyond chance between the VCS image-read and manually-read LBC results.

Table S3.2 shows the findings for the DHM re-read in relation to the original VCS reading. The (weighted) percentage of agreement between the original VCS image-read and the QC DHM image-read re-read LBC results was 96.0%. The weighted kappa-statistic measure of agreement was estimated to be 0.56, representing moderate agreement beyond chance between the VCS image-read and the DHM image-read LBC results. When samples with discordant results were tested for HPV, none were found to be HPV16/18 positive while 12 were found to be positive for other high-risk HPV.

For clinical management purposes, reports were mended when, based on the result of the re-testing procedure, an earlier follow-up was required. Of the 911 participants originally given a 2.5 year recall allocation for a routine screening visit, 8 (0.8% of the overall number of samples) were amended to possible/definite low grade abnormalities (ASCUS/LSIL) and will be recalled for a follow-up surveillance visit in 12 months. Two (0.2% of the overall number of samples) were amended to possible high grade abnormalities (ASC-H) and referred to colposcopy. At colposcopy, one of these woman had a histological diagnosis of CIN1 and the second women had a negative (normal) biopsy result. Table S3.3 provides a summary of the clinical

management changes. Note that these changes were not considered in the analysis of data for the purposes of the study.

Table A. Dis/agreement counts and percentages for the VCS image-read results (original) and manually-read LBC results (re-read).

	VCS Manual-Read Result (re-reading)						
**Original VCS Image-Read Result**	**NEGATIVE**	**ASCUS**	**LSIL**	**ASC-H**	**HSIL**	**UNSAT**	**Total**
**NEGATIVE**	893<br>87.2%	8<br>0.8%	3<br>0.3%	3<br>0.3%	0<br>0.0%	4<br>0.4%	911<br>89.0%
**ASCUS**	2<br>0.2%	27<br>2.6%	6<br>0.6%	2<br>0.2%	0<br>0.0%	1<br>0.1%	38<br>3.7%
**LSIL**	0<br>0.0%	6<br>0.6%	23<br>2.2%	1<br>0.1%	0<br>0.0%	1<br>0.1%	31<br>3.0%
**ASC-H**	0<br>0.0%	0<br>0.0%	0<br>0.0%	1<br>0.1%	0<br>0.0%	0<br>0.0%	1<br>0.1%
**HSIL**	0<br>0.0%	0<br>0.0%	0<br>0.0%	0<br>0.0%	1<br>0.1%	0<br>0.0%	1<br>0.1%
**UNSAT**	2<br>0.2%	0<br>0.0%	0<br>0.0%	0<br>0.0%	0<br>0.0%	40<br>3.9%	42<br>4.1%
**Total**	897<br>87.6%	41<br>4.0%	32<br>3.1%	7<br>0.7%	1<br>0.1%	46<br>4.5%	1,024<br>100.0%

Table B. Dis/agreement count and percentages for the VCS image-read (original reading) and DHM image-read LBC (QC re-read) results.

	DHM Image-Read Result							
**VCS Image-Read Result**	**NEGATIVE**	**ASCUS**	**LSIL**	**ASC-H**	**HSIL**	**AGCUS**	**UNSAT**	**Total**
**NEGATIVE**	890<br>86.9%	12<br>1.2%	2<br>0.2%	3<br>0.3%	0<br>0.0%	1<br>0.1%	3<br>0.3%	911<br>89.0%
**ASCUS**	30<br>2.9%	4<br>0.4%	2<br>0.2%	1<br>0.1%	0<br>0.0%	0<br>0.0%	1<br>0.1%	38<br>3.7%
**LSIL**	16<br>1.6%	4<br>0.4%	11<br>1.1%	0<br>0.0%	0<br>0.0%	0<br>0.0%	0<br>0.0%	31<br>3.0%
**ASC-H**	0<br>0.0%	0<br>0.0%	0<br>0.0%	1<br>0.1%	0<br>0.0%	0<br>0.0%	0<br>0.0%	1<br>0.1%
**HSIL**	0<br>0.0%	0<br>0.0%	0<br>0.0%	0<br>0.0%	1<br>0.1%	0<br>0.0%	0<br>0.0%	1<br>0.1%
**UNSAT**	20<br>2.0%	0<br>0.0%	0<br>0.0%	0<br>0.0%	0<br>0.0%	0<br>0.0%	22<br>2.1%	42<br>4.1%
**Total**	956<br>93.4%	20<br>2.0%	15<br>1.5%	5<br>0.5%	1<br>0.1%	1<br>0.1%	26<br>2.5%	1,024<br>100.0%

*Atypical glandular cells of uncertain significance

Table C. Dis/agreement count and percentages between the recommendations made on the basis of the original VCS image-read LBC results and those based on the results of the independent laboratory QC re-read.

	Recommendation after retesting				
**Original recommendation**	**Routine screening**	**1 year surveillance follow-up**	**Colposcopy**	**Repeat testing**	**Total**
**Routine screening**	901<br>88.0%	8<br>0.8%	2<br>0.2%	0<br>0.0%	911<br>89.0%
**1 year surveillance follow-up**	0<br>0.0%	69<br>6.7%	0<br>0.0%	0<br>0.0%	69<br>6.7%
**Colposcopy**	0<br>0.0%	0<br>0.0%	2<br>0.2%	0<br>0.0%	2<br>0.2%
**Repeat testing**	0<br>0.0%	0<br>0.0%	0<br>0.0%	42<br>4.1%	42<br>4.1%
**Total**	904<br>88.0%	77<br>7.5%	4<br>0.4%	42<br>4.1%	1027<br>100.0%

Discussion and conclusions

The variation of results identified by the QC re-read is consistent with the level of inter-laboratory variability commonly observed in the program. The trial Independent Data and Safety Monitoring Committee (IDSMC) reviewed these findings and concluded that the original LBC reading was appropriate, therefore the main findings reported in the trial analysis are based on the original LBC reading.
