# Supplementary material for: Cervical screening with primary HPV testing or cytology in a population of women in which those aged 33 years or younger had previously been offered HPV vaccination: Results of the Compass pilot randomised trial
Source: PLoS Med. 2017 Sep 19;14(9):e1002388. doi: 10.1371/journal.pmed.1002388 (PMC5604935; doi:10.1371/journal.pmed.1002388)
Supplement: S4 Text — (PDF) [file pmed.1002388.s004.pdf]

Appendix to the manuscript:

Cervical screening with primary HPV testing or cytology in a population of women in which those aged 33 years or younger were offered vaccination: Results of the Compass pilot randomized trial

Karen Canfell,[1,2,3*] Michael Caruana, [1] Val Gebski, [4] Jessica Darlington-Brown MPH, [1] Stella Heley, [5] Julia Brotherton, [5,6] Dorota Gertig, [5,6,] Chloe J. Jennett, [1] Annabelle Farnsworth, [2,7] Jeffrey Tan, [8,9] C. David Wrede, [8,9] Philip E. Castle, [10] Marion Saville, [5,6]

[1]Cancer Research Division, Cancer Council New South Wales, Sydney, New South Wales, Australia

[2]School of Public Health, Sydney Medical School, University of Sydney, Sydney, New South Wales, Australia

[3]Prince of Wales Clinical School, The University of New South Wales, Sydney, New South Wales, Australia

[4]NHMRC Clinical Trials Centre, University of Sydney, Sydney, New South Wales, Australia

[5]Victorian Cytology Service Ltd., Melbourne, Victoria, Australia *(affiliation during time of the work for DG)*

[6]School of Public Health, University of Melbourne, Melbourne, Victoria, Australia

[7]Douglas Hanly Moir Laboratory, Sydney, New South Wales, Australia

[6]Department of Obstetrics and Gynaecology, University of Melbourne, Melbourne, Victoria, Australia

[9]Department of Oncology & Dysplasia, The Royal Women's Hospital, Melbourne, Victoria, Australia

[10]Albert Einstein College of Medicine, Bronx, New York, The United States of America.

Appendix Section S4.

Adverse Event Report

Table S4 provides details on the cumulative number of adverse events by study arm, to August 31st 2016. All events were reviewed by the trial Independent Data and Safety Monitoring Committee (IDSMC) and reported to the ethics committee as required.

Four deaths occurred in study participants during follow-up - these events were reviewed by the IDSMC who confirmed that due to the unrelated nature of the events the trial should proceed without modification. The two reported adverse events in Table 1 did not relate to clinical outcomes but to an issue with reporting the screening result to the participant which was resolved. No invasive cervical cancer cases stage Ia2 and above were reported in follow-up (it should be noted that one Stage 1a1 microinvasive cancer was detected via the screening episode in the HPV+LBC triage group, but this is not an adverse event since the invasive cancer was appropriately screen-detected).

Table D. Summary of Adverse Events*

	**Study Arm 1**	**Study Arm 2**	**Study Arm 3**
Deaths in follow-up (classified as unrelated to the trial)	0	2 (0.1%)	2 (0.1%)
Reported Serious Adverse Event (excluding deaths)	0	0	0
Reported Adverse Event	1 (0.1%)	1 (0.05%)	0

*No adverse events were reported in participants who withdrew from the study.
