# Supplementary material for: Cervical screening with primary HPV testing or cytology in a population of women in which those aged 33 years or younger had previously been offered HPV vaccination: Results of the Compass pilot randomised trial
Source: PLoS Med. 2017 Sep 19;14(9):e1002388. doi: 10.1371/journal.pmed.1002388 (PMC5604935; doi:10.1371/journal.pmed.1002388)
Supplement: S6 Text — (PDF) [file pmed.1002388.s006.pdf]

TheAlfred

ETHICS COMMITTEE CERTIFICATE OF APPROVAL

This is to certify that

Project No: HREC/13/Alfred/1 (Local Reference: Project 111/13)

Project Title: Compass Trial Pilot Study: A pilot for a randomised controlled trial of primary HPV DNA testing for cervical cancer screening in Australia

Principal Researcher: Professor Marion Saville

was considered under the Consultative Council for Clinical Trial Research (CCCTR) Streamlined Ethics Review Program (SERP) by the Ethics Committee on ***26-Mar-2013****, meets the requirements of the National Statement on Ethical Conduct in Human Research (2007) and was* ***APPROVED*** *on* ***30-Aug-2013***

It is the Principal Researcher's responsibility to ensure that all researchers associated with this project are aware of the conditions of approval and which documents have been approved.

The Principal Researcher is required to notify the Secretary of the Ethics Committee, via amendment or progress report, of

- Any significant change to the project and the reason for that change, including an indication of ethical implications (if any);
- Serious adverse effects on participants and the action taken to address those effects;
- Any other unforeseen events or unexpected developments that merit notification;
- The inability of the Principal Researcher to continue in that role, or any other change in research personnel involved in the project;
- Any expiry of the insurance coverage provided with respect to sponsored clinical trials and proof of re-insurance;
- A delay of more than 12 months in the commencement of the project; and,
- Termination or closure of the project.

Additionally, the Principal Researcher is required to submit

- A Progress Report on the anniversary of approval and on completion of the project *(forms to be provided)*;

The Ethics Committee may conduct an audit at any time.

All research subject to the Alfred Hospital Ethics Committee review must be conducted in accordance with the National Statement on Ethical Conduct in Human Research (2007).

The Alfred Hospital Ethics Committee is a properly constituted Human Research Ethics Committee in accordance with the National Statement on Ethical Conduct in Human Research (2007).

SPECIAL CONDITIONS

Response to Ethics Committee dated 20-Mar-2013 regrading revisions to the Protocol to be incorporated into the revised Protocol.

Approved documents

Documents reviewed and approved at the meeting were:

Document	*Version*	*Date*
Protocol: Compass	1.5	12 February 2013
Master Participant Information & Consent Sheet	1.3	19 March 2013
Master Consent Form	1.3	19 March 2013
Compass Trial Details for Practitioners		06 March 2013
Cover Letter to GPs		06 March 2013
Reference List		06 March 2013
Poster	1.1	28 February 2013

Approved Sites:
Approval is given for this research project to be conducted at the following sites and campuses:
Victorian Cytology Service
Alfred Health –Melbourne Sexual Health Centre
Royal Women's Hospital

Site-Specific Assessment (SSA)
SSA authorisation is required at all sites participating in the study. SSA must be authorised at a site before the research project can commence.

The completed Site-Specific Assessment Form and a copy of this ethics approval letter must be submitted to the Research Governance Officer for authorisation by the Chief Executive or delegate. This applies to each site participating in the research.

The HREC wishes you and your colleagues every success in your research.

I.K.D

K Dunscombe
Acting Secretary, Ethics Committee

Please quote project number and title in all correspondence
